# Optimal Detecting Part of *Ophiocordyceps sinensis* for Identifying Wild and Cultivated Categories Using Hyperspectrum

**DOI:** 10.3390/s26175672

**Published:** 2026-09-07

**Authors:** Shihao Xie, Xingfeng Chen, Hejuan Du, Jiaguo Li, Dawa Zhuoma, Jun Liu, Limin Zhao, Jianwei Wang, Shu Liu

**Affiliations:** 1 School of Computer Science and Engineering, University of Emergency Management, Langfang 065201, China; xiesh@st.cidp.edu.cn (S.X.); lijg@aircas.ac.cn (J.L.); 2 Aerospace Information Research Institute, Chinese Academy of Sciences, Beijing 100094, China; liujun02@aircas.ac.cn (J.L.); zhaolm@aircas.ac.cn (L.Z.); wangjw@aircas.ac.cn (J.W.); 3 School of Cyberspace Security, Xizang Minzu University, Xianyang 712089, China; 4 Xizang Autonomous Region Key Laboratory of Quality Research of Tibetan Medicine, Xizang Institute for Food and Drug Control, Lhasa 850000, China; dazhuo1108@163.com; 5 Jilin Provincial Key Laboratory of Chinese Medicine Chemistry, Changchun Institute of Applied Chemistry, Chinese Academy of Sciences, Changchun 130022, China; mslab20@ciac.ac.cn

**Keywords:** hyperspectral, *Ophiocordyceps sinensis*, machine learning, optimal detecting part

## Abstract

Hyperspectral technology has become an important method for identifying wild and cultivated *Ophiocordyceps sinensis*, but existing studies mainly focus on intact samples. In the actual circulation process, *Ophiocordyceps sinensis* often breaks, and samples missing the stroma part but retaining the larva part still possess practical identification value. However, accommodating the identification of both intact samples and stroma-missing samples requires clarifying the difference in spectroscopic identification information contribution between the larva part and the stroma part, thereby optimizing the data acquisition strategy for specialized *Ophiocordyceps sinensis* detection instruments. Based on the segmentation of hyperspectral images of intact *Ophiocordyceps sinensis*, this study constructed simulated single and mixed part datasets for machine learning model training to analyze the spectral feature differences of different parts. Furthermore, real stroma-missing samples were used to verify the identification capability of the models in the practical scene. Finally, the optimal detecting part was determined by comprehensively considering the highest identification accuracy and the maximum application scope. Both the larva part and the stroma part exhibit effective spectroscopic identification information contribution for identifying wild and cultivated *Ophiocordyceps sinensis*. However, the spectroscopic identification information contribution of the larva part is greater than that of the stroma part. Multiple comparative experimental results show that the model trained with intact samples can be directly used for the identification of stroma-missing *Ophiocordyceps sinensis* with the highest accuracy of 98.52%. This conclusion provides a basis for the practical application of hyperspectral technology in *Ophiocordyceps sinensis*.

## 1. Introduction

*Ophiocordyceps sinensis* is a traditional Chinese medicine with high medicinal and economic value, exhibiting multiple biological activities such as anti-aging [1], anti-inflammatory [2], and antioxidant [3]. Although widely perceived as a Chinese medicine, *Ophiocordyceps sinensis* is also used as a functional food [4]. In recent years, with the gradual decrease of wild resources and the continuous growth of market demand, the cultivated *Ophiocordyceps sinensis* has achieved large-scale production [5,6,7]. However, due to the high morphological similarity between wild and cultivated products [8], cultivated products are used to pass off as the expensive wild ones in the market, imposing higher demands on the quality control and market supervision of *Ophiocordyceps sinensis* [9].

Currently, the identification methods for *Ophiocordyceps sinensis* mainly include morphological identification, microscopic identification, chemical composition analysis, and molecular biology analysis, yet all possess varying degrees of limitations. The morphological-characteristic-based method, relying on the professional’s subjective judgment and practical experience, has traditionally long been controversial on account of the lack of objective standards [10]. Microscopic identification observes microstructures such as mycelium, but it typically requires sectioning, causing a certain degree of destructiveness to the samples [11,12]. Chemical composition analysis (e.g., HPLC, LC-MS/MS) achieves identification by detecting characteristic components such as adenosine, cordycepin, and polysaccharides, yielding high accuracy [9,13,14]; however, the detection process relies on complex sample pretreatment, expensive instruments, and specialized technicians, resulting in a long detection cycle. Molecular biology methods (e.g., DNA analysis) can identify *Ophiocordyceps sinensis* at the genetic level with high species recognition capability [15], but they similarly face issues such as complex procedures, high experimental costs, and difficulties in achieving rapid on-site detection.

Hyperspectral technology has been increasingly applied to the quality evaluation and identification of traditional Chinese medicines for rapid, non-destructive measurements while capturing chemical and structural information across broad spectral ranges [16,17,18,19]. Zhao et al. used hyperspectral reflectance data and random forest (RF) to identify *ginseng* growth years and reported independent-test accuracies of 92.9% and 100% for different food and medicine age boundaries, while also identifying the spectral regions with the greatest discriminatory importance [20]. Chen et al. integrated spectral and spatial features in an fully connected-convolutional neural network (FC-CNN) framework for *Panax ginseng* and reported 100% accuracy for food and medicinal purposes [21]. For *Radix glycyrrhizae*, Yan et al. evaluated Vis/NIR and NIR hyperspectral imaging with logistic regression (LR), support vector machine (SVM), convolutional neural network (CNN), and recurrent neural network (RNN) models for geographical origin identification and reported accuracies above 90% across the evaluated calibration, validation, and prediction sets [22]. These studies demonstrate that hyperspectral technology can capture subtle differences associated with origin, growth stage, and quality status.

For *Ophiocordyceps sinensis*, previous analytical studies have also established that wild and cultivated categories possess differences. NIRS combined with model transfer techniques has been used for non-destructive discrimination of wild and cultivated *Ophiocordyceps sinensis*, with external test accuracy exceeding 95% [23]. More recently, label-free SERS coupled with machine learning (ML) models achieved 98.95% accuracy and 98.38% five-fold cross-validation accuracy for identifying wild and cultivated *Ophiocordyceps sinensis*, while metabolomics was used to interpret the spectral differences [24]. Du et al. investigated four specimen orientations and found no statistically significant difference in identification performance, with LR achieving an average accuracy above 98% [25]. This result is important for instrument design because it suggests that strict orientation control may be unnecessary. Nevertheless, it does not determine whether the different parts exhibit meaningful differences in spectroscopic identification information contribution.

**Figure 1:**
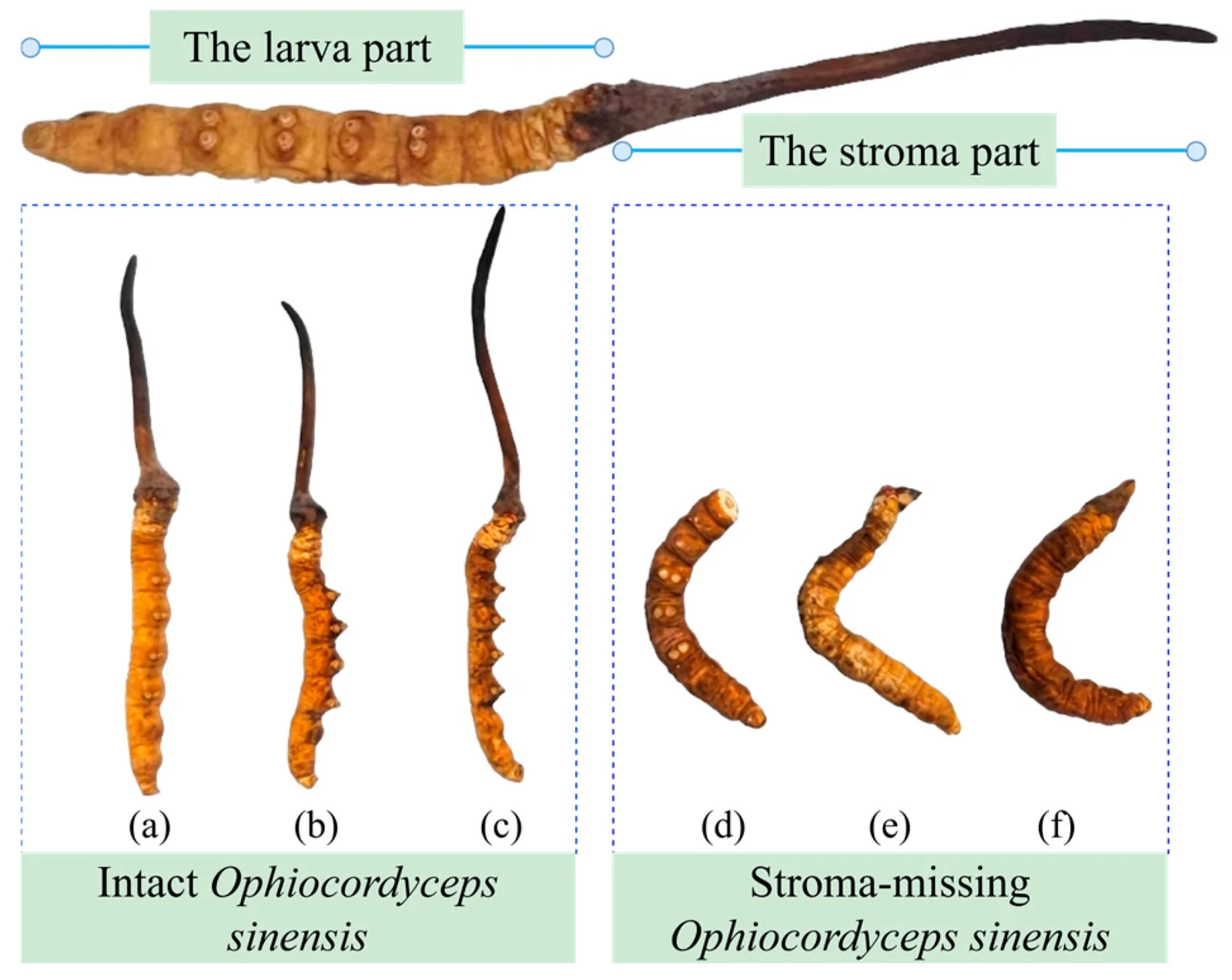
The different parts of *Ophiocordyceps sinensis*: (**a**–**c**) are intact *Ophiocordyceps sinensis*; (**d**–**f**) are stroma-missing *Ophiocordyceps sinensis*.

*Ophiocordyceps sinensis*, based on morphological characteristics, is composed of two parts: the larva (i.e., *mummified* caterpillar) and the stroma (i.e., fungal fruiting body) [5]. Existing hyperspectral identification studies usually conduct model training and validation based on morphologically intact samples. However, in the actual excavation, transportation, and circulation processes, the breakage or detachment of the stroma part is very common, forming a large number of samples that lack the stroma part but maintain the larva part. Because the healing power of *Ophiocordyceps sinensis* is believed to be concentrated in the caterpillar (i.e., the larva part), which is indeed filled with *Ophiocordyceps sinensis* mycelium [4], such samples maintain market value and practical identification demand. Nevertheless, the lack of the stroma part alters the sample morphology, which may cause changes in spectral features, placing identification models built on intact samples at risk of a significant decline in identification accuracy in real application scene. Figure 1 illustrates the different parts of *Ophiocordyceps sinensis*.

In the process of developing specialized detection instruments for hyperspectral identification, sample morphological differences affect and constrain the design of instrument detecting methods and the operational procedures, which need to specify the orientation or detecting part. *Ophiocordyceps sinensis* is asymmetrical in the orientation dimension, possessing different orientations such as the lateral, dorsal, and ventral. Du et al. have confirmed that the orientation of *Ophiocordyceps sinensis* has a minor impact on model identification [25] and that the identification accuracy for wild and cultivated *Ophiocordyceps sinensis* across all orientations exceeds 98%, thus the detecting orientation does not require restriction. From another dimension, *Ophiocordyceps sinensis* is also asymmetrical in terms of parts, possessing both the larva and the stroma parts, which exhibit significant differences in appearance and biological composition. For parts with obvious spectroscopic identification information contribution, specialized hyperspectral detection instruments must acquire the data; for parts contributing inconspicuous spectroscopic identification information, adding a physical occlusion device to prevent data acquisition may even need to be considered. However, whether it is necessary to add a part-occlusion device depends on the respective contribution degrees of the larva and stroma parts to the identification information, and there is currently no systematic study discussing this issue. Therefore, prior to the engineering application of specialized identification instruments for *Ophiocordyceps sinensis*, two key questions still need to be answered.

The first is a qualitative question: For *Ophiocordyceps sinensis*, do differences exist in the spectroscopic identification information contributions of the intact, larva, and stroma parts for identifying wild and cultivated categories? If there is no difference in the spectroscopic identification information contributions of each part, there is no need to restrict the detecting part; if differences exist, it is necessary to further clarify which part provides spectral information that yields higher identification accuracy.

The second is a quantitative question: If differences in the spectroscopic identification information contributions exist among parts, how extensive are these differences? Can they guarantee that specialized hyperspectral detection instruments possess high-accuracy detection capabilities for stroma-missing *Ophiocordyceps sinensis*, which is widely present in the real market scene? If the difference is large, parts with low contributions should not be observed to ensure high accuracy; if the difference is small, mixed inputs of different parts can be accepted to expand the application scope of the specialized detection instruments.

Based on the above two questions, utilizing the identification of wild and cultivated *Ophiocordyceps sinensis* as the research task, this study collected data using hyperspectral technology. Simulated single and mixed part datasets were constructed using the hyperspectral image segmentation of intact *Ophiocordyceps sinensis*. The larva and stroma parts were distinguished, and spectral reflectance data of different parts were obtained through averaging processing. Subsequently, LR, SVM, RF, XGBoost, LightGBM and fully connected neural network (FCNN) models were trained on these datasets to determine whether the identification performance differed among different parts. The identification performance of these models on real stroma-missing *Ophiocordyceps sinensis* samples was then evaluated, and the differences in identification performance among models trained using different parts were quantitatively analyzed.

The main contributions of this study are as follows: a qualitative comparison of the spectroscopic identification information contribution by the intact, larva, and stroma parts for identifying wild and cultivated *Ophiocordyceps sinensis*; the validation of the trained models using 135 real market samples with missing stroma; and the identification of the optimal detecting part to guide the design of specialized hyperspectral identification instruments.

## 2. Methodology

### 2.1. Strategy

**Figure 2:**
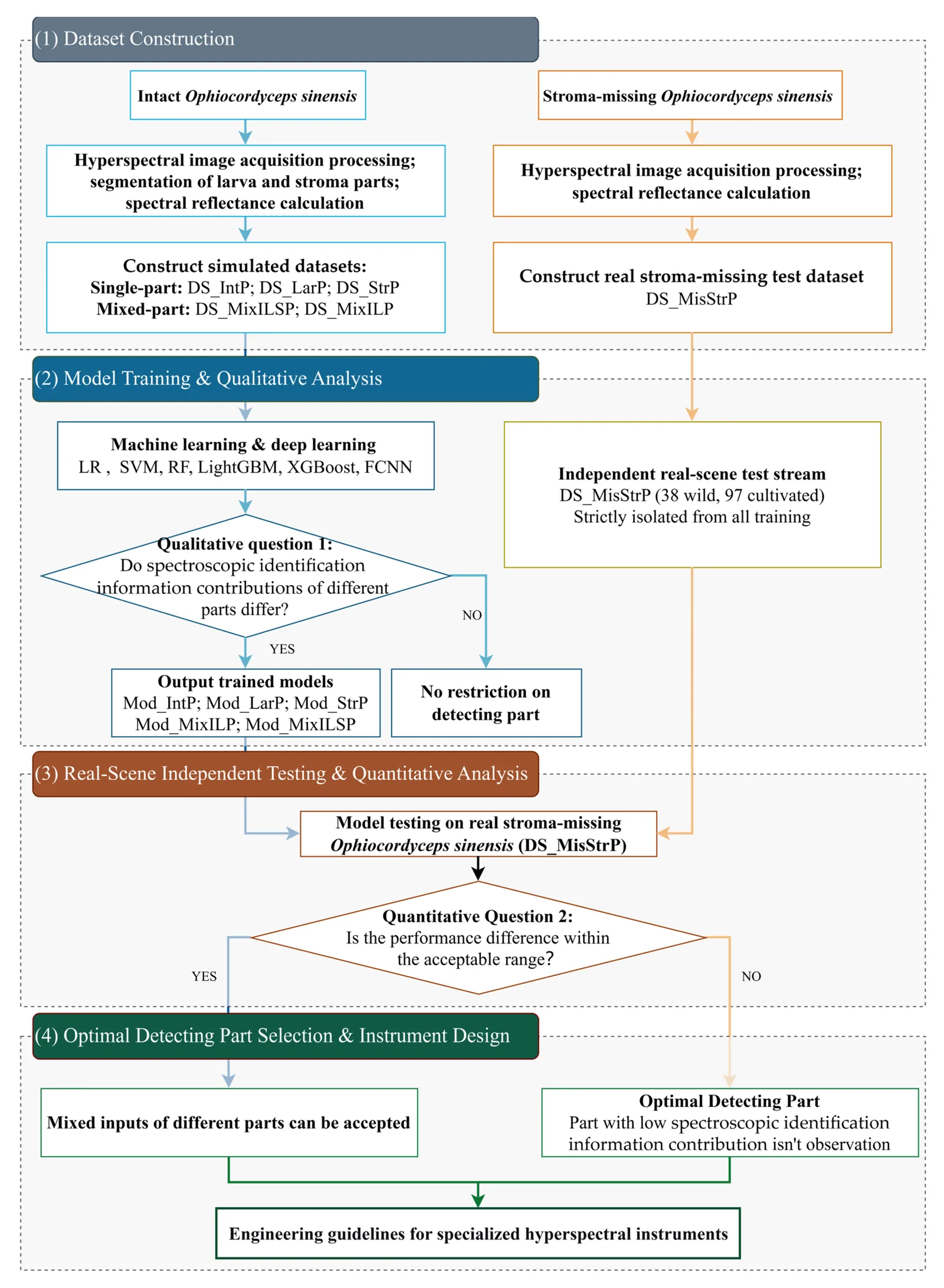
Flowchart of designing an optimal detecting part for the hyperspectral identification of *Ophiocordyceps sinensis*.

This study focuses on the detecting part in the engineering application of hyperspectral technology for *Ophiocordyceps sinensis*, focusing on answering two questions. First, whether there is a difference in the contribution of spectral identification information among the larva part, stroma part, and intact part. Second, if a difference exists, whether this difference can be identified in stroma-missing samples in the real scene, and how to optimize the detecting part of the instrument accordingly. The entire research workflow, comprising four steps, is shown in Figure 2.

(1) Dataset Construction

Based on the hyperspectral images of intact *Ophiocordyceps sinensis*, the larva part and the stroma part were obtained through manual visual image segmentation, and their spectral information was respectively extracted to construct simulated datasets, including the intact part dataset (DS_IntP), the larva part dataset (DS_LarP), and the stroma part dataset (DS_StrP). To simulate the appearance of broken *Ophiocordyceps sinensis* (i.e., retaining only the larva part or the stroma part, thus only considering the remaining parts to be intact) during the actual application process, two types of mixed part datasets were further constructed based on the above datasets: a mixed dataset of intact, larva, and stroma parts (DS_MixILSP), and a mixed dataset of intact and larva parts (DS_MixILP). In addition, real stroma-missing samples circulating in the market were collected to construct an independent test dataset (DS_MisStrP) for subsequent real-scene testing.

DS_MixILP simulates a scene where intact *Ophiocordyceps sinensis* and stroma-missing *Ophiocordyceps sinensis* are mixed, which is used to simulate the situation where both exist in the market and require identification, to study the applicability of the model, and to further evaluate the difference between the intact part and the larva part. DS_MixILSP simulates a scene where intact *Ophiocordyceps sinensis* and broken *Ophiocordyceps sinensis* are mixed; *Ophiocordyceps sinensis* with only the stroma part remaining possess low value and lack identification significance, thus constructing this dataset is to form a comparison with DS_MixILP to study the impact of the stroma part on identification.

(2) Model Training

Five simulated datasets were used to train LR, SVM, RF, XGBoost, LightGBM and FCNN models, respectively. Among these, by comparing the identification performance of models trained on DS_IntP, DS_LarP, and DS_StrP, the spectroscopic identification information contribution of the intact, larva, and stroma parts for identifying wild and cultivated *Ophiocordyceps sinensis* were analyzed. Furthermore, models trained using DS_MixILSP and DS_MixILP were utilized to evaluate the impact on identification stability when different parts were randomly mixed as model inputs, thereby determining the robustness of the models to different parts.

To conduct independent test dataset in the real scene, 1–2 models with the best performance in the aforementioned simulation experiments were selected. All sample data from DS_IntP, DS_LarP, DS_StrP, DS_MixILSP, and DS_MixILP datasets were utilized as training datasets to train and save the models. The respective trained models were named as follows: Mod_IntP, Mod_LarP, Mod_StrP, Mod_MixILSP, and Mod_MixILP, with the corresponding suffix added for each specific model.

(3) Real-scene testing

DS_MisStrP was used as an independent test dataset to validate the various models obtained in the previous step (Mod_IntP, Mod_LarP, Mod_StrP, Mod_MixILSP, and Mod_MixILP). By comparing the identification accuracy of models trained on different datasets against real broken samples, the magnitude of the identification difference among different parts was quantitatively evaluated. The question of whether the part contribution rules obtained from the simulation experiments could be applied to the real application scene was also analyzed.

(4) Optimal Detecting Part Selection

Comprehensively considering the simulation data analysis results and the real scene test results, different detecting part strategies were evaluated from the two aspects of model identification accuracy and engineering application scope. If the highest identification accuracy is the target, the intact sample is prioritized as the detecting part; if the practical application requirements for both intact samples and stroma-missing samples need to be accommodated, the performance of the mixed part training strategy is further analyzed. On this basis, the optimal detecting part scheme suitable for hyperspectral identification instruments for *Ophiocordyceps sinensis* is proposed, providing a basis for designing the data acquisition strategy of the instrument.

### 2.2. Data

#### 2.2.1. Sample Acquisition

A total of 1052 *Ophiocordyceps sinensis* samples were used in the study and these were divided into two categories: 627 wild samples and 425 cultivated samples. This included 917 intact *Ophiocordyceps sinensis* and 135 stroma-missing *Ophiocordyceps sinensis*. Specific quantity distributions are detailed in Table 1.

The collected samples were obtained from geographic origins by personnel dispatched from the Xizang Institute for Food and Drug Control. Xizang, a provincial autonomous region in China, is a major production area for wild *Ophiocordyceps sinensis* [26], located in the southwest of the Tibetan Plateau. The wild samples in this study are all from this region. The differences in altitude, climate, and local ecology in these areas represent a range of sampling environments within Xizang and among wild populations. The cultivated *Ophiocordyceps sinensis* samples were purchased from dealers of multiple commercial brands across various production areas such as Qinghai, Zhejiang, and Sichuan. Although cultivated *Ophiocordyceps sinensis* samples originate from various regions and commercial brands, they exhibit a certain degree of uniformity. At present, the primary host insect used in large-scale artificial cultivation is predominantly *Thitarodes xiaojinensis*, and the infecting fungus employed is the anamorph of *Ophiocordyceps sinensis*, namely *Hirsutella sinensis* [27]. The entire cultivation process is conducted under standardized, artificially controlled environmental conditions, which differ markedly from those of wild *Ophiocordyceps sinensis*.

After collection, all samples were cleared of surface dust and loose debris but did not undergo further processing. The entire sample acquisition process was strictly standardized to ensure that the obtained conclusions are based on a comprehensive and authentic dataset.

#### 2.2.2. Hyperspectral Image Acquisition

Hyperspectral image acquisition was completed using two push-broom hyperspectral cameras manufactured by NEO company in Oslo, Norway, including Hyspex VNIR-1024 (400–1000 nm) and Hyspex SWIR-384 (930–2500 nm). The two systems jointly performed continuous hyperspectral data collection from the samples across the 400–2500 nm wavelength range. Table 2 lists the specific parameters of the two hyperspectral cameras.

The imaging system was installed in a darkroom and operated at ambient laboratory temperature under controlled illumination conditions. Both cameras were mounted on a rigid imaging frame above a motorized translation stage for line-scan image acquisition. The 30 cm lens configuration was selected for both the VNIR and SWIR cameras. Illumination was provided by two 100 W halogen lamps symmetrically positioned on both sides of the sample, with an incidence angle of 22.5° relative to the sample surface normal. The lamps were warmed up for at least 30 min before image acquisition to ensure stable illumination conditions.

**Figure 3:**
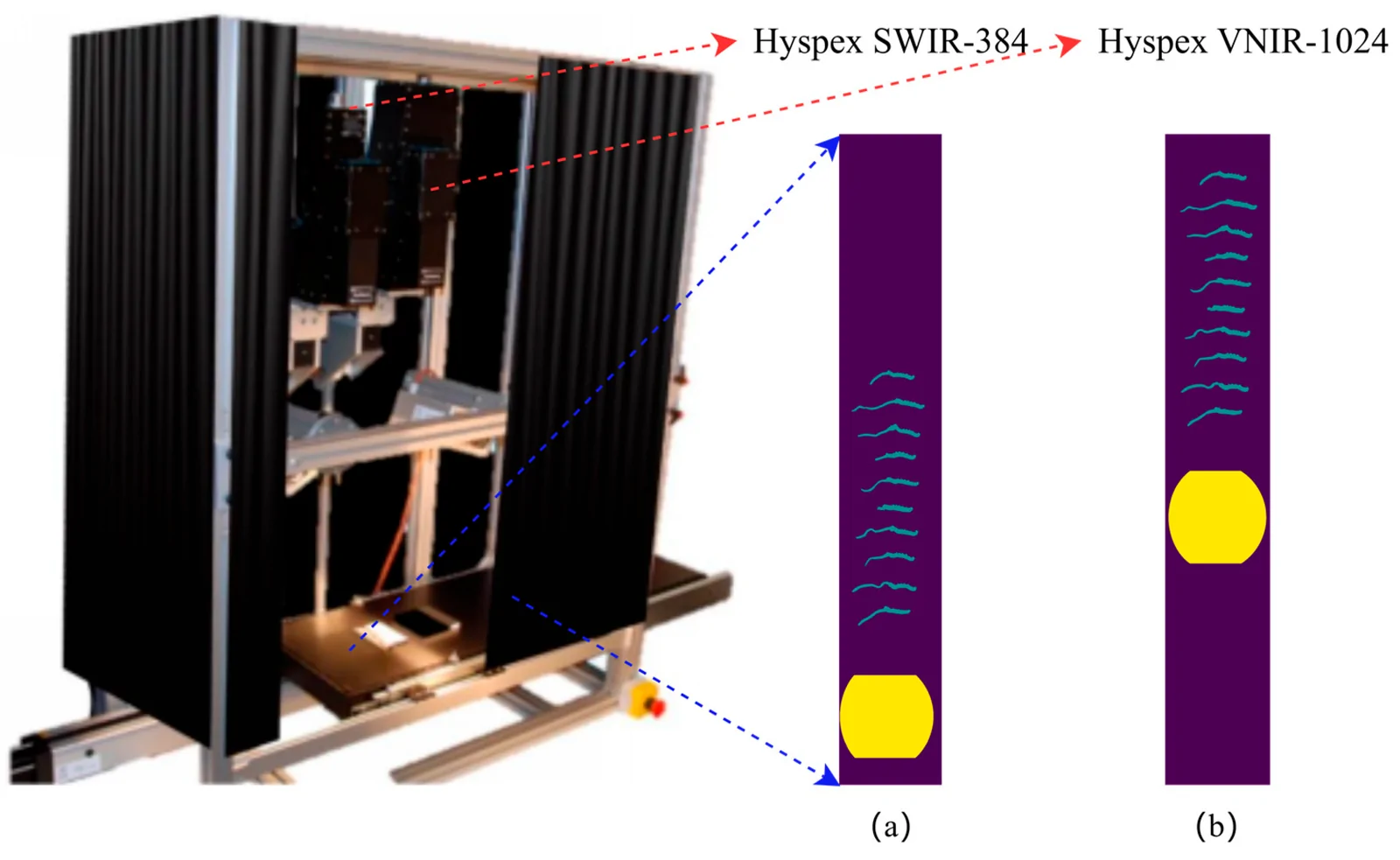
The assembly environment of the hyperspectral cameras and the false-color composite images of the collected samples. Red dashed arrows indicate the positions of the Hyspex SWIR-384 and VNIR-1024 cameras; blue dashed arrows indicate the corresponding scanning areas of the samples. (**a**) and (**b**) are the false-color composite images acquired by the Hyspex SWIR-384 and VNIR-1024 cameras, respectively, where green regions represent *Ophiocordyceps sinensis* samples and yellow regions represent the whiteboard.

The integration times were set to 4.0 ms for the VNIR camera and 4.5 ms for the SWIR camera, with corresponding frame periods of 19.0 ms and 49.535 ms, respectively. During image acquisition, the SWIR focal plane array (FPA) was maintained at 150 K. During acquisition, 200 background frames were recorded for each camera for subsequent dark current correction. The samples were placed on the motorized translation stage and scanned sequentially under the VNIR and SWIR cameras. A standard whiteboard was simultaneously placed during each acquisition for reflectance correction. Figure 3 is the assembly environment of the hyperspectral cameras and the false-color composite images of the collected samples.

The acquired hyperspectral images contained multiple *Ophiocordyceps sinensis* samples together with a corresponding whiteboard within the same field of view. Therefore, a hyperspectral data processing procedure was established, including each sample and whiteboard extraction from the original hyperspectral images; image conversion from digital number (DN) to surface reflectance; segmentation of the larva part and stroma part; and the mean reflectance calculation of each spectral band.

To extract individual samples and the whiteboard from the original hyperspectral images, a single-band thresholding method was applied to binarize the grayscale images of the VNIR and SWIR images at 956 nm and 1230 nm, respectively, thereby generating binary mask images. These two wavelengths provided high contrast between the target objects and the background. The mask image was subsequently refined using morphological connected component analysis to remove isolated noise, eliminate small connected regions, and crop the upper and lower boundaries of the whiteboard to suppress reflection and shadow artifacts occurring at the interface between the whiteboard and the conveyor belt, with the optimized mask image shown in Figure 3. Finally, connected component analysis was performed to separate individual samples within each mask image. Each connected component was regarded as an independent *Ophiocordyceps sinensis* region of interest (OROI), whereas the extracted whiteboard in the same image was defined as the whiteboard region of interest (WROI).

During radiometric correction, column-wise normalization was adopted to address variations in illumination intensity and detector response across different image columns of the push-broom hyperspectral imaging system. Specifically, for each band, all DN values of OROI were corrected, using the WROI in the same field of view as a reference, and dividing each column by the mean value of WROI to obtain the reflectance values of OROI. The reflectance calculation is as follows:

(1) $$\rho_{\lambda \left(i,j\right)}=\frac{DN_{\left(i,j\right)}}{E\left(DN_{wb\left(:,j\right)}\right)}$$

where $\rho_{\lambda \left(i,j\right)}$ is the reflectance value of OROI of the i-th row and j-th column under λ, λ is the wavelength, $DN_{\left(i,j\right)}$ is the pixel DN of the i-th row and j-th column before correction, and $E\left(DN_{wb\left(:,j\right)}\right)$ is the mean value of all pixel DN in the j-th column of WROI.

Following radiometric calibration, each intact *Ophiocordyceps sinensis* sample was further divided into the larva part and the stroma part. The segmented data form a simulation of the broken *Ophiocordyceps sinensis* in the real scene. An interactive artificial segmentation method was adopted in which the boundary between the larva and the stroma parts was delineated according to their natural anatomical junction. Referring to Figure 1, the segmentation boundary was defined at the transitional zone where the color gradually shifts from light to dark along the direction from the larva to the stroma (as exemplified by *Ophiocordyceps sinensis* sample (e) in Figure 1). All manual segmentation procedures were performed by a single operator to ensure operational consistency. The OROI of each intact sample was partitioned into the larva part region of interest (LROI) and the stroma part region of interest (SROI).

After radiometric calibration, the hyperspectral reflectance data of each sample consisted of 108 VNIR bands and 288 SWIR bands, yielding a total of 396 spectral bands. For each band, the mean reflectance within the corresponding ROI was calculated. Specifically, the mean reflectance of each single-band OROI was calculated using Equation (2), and the same procedure was applied to the LROI and SROI. Thus, 396 mean reflectance values were obtained for each ROI. These values were then arranged according to wavelength to generate the hyperspectral reflectance curve for each ROI. The mean reflectance of a single-band OROI was calculated as follows:

(2) $$\rho_{\lambda I}=\frac{\sum \rho_{\lambda \left(i,j\right)}}{N_{OROI}}$$

where $\rho_{\lambda I}$ is the mean reflectance of a single-band OROI image with wavelength λ, $\sum \rho_{\lambda \left(i,j\right)}$ is the sum of the reflectance of all OROI pixels at wavelength λ in the image, and $N_{OROI}$ is the number of OROI pixels.

**Figure 4:**
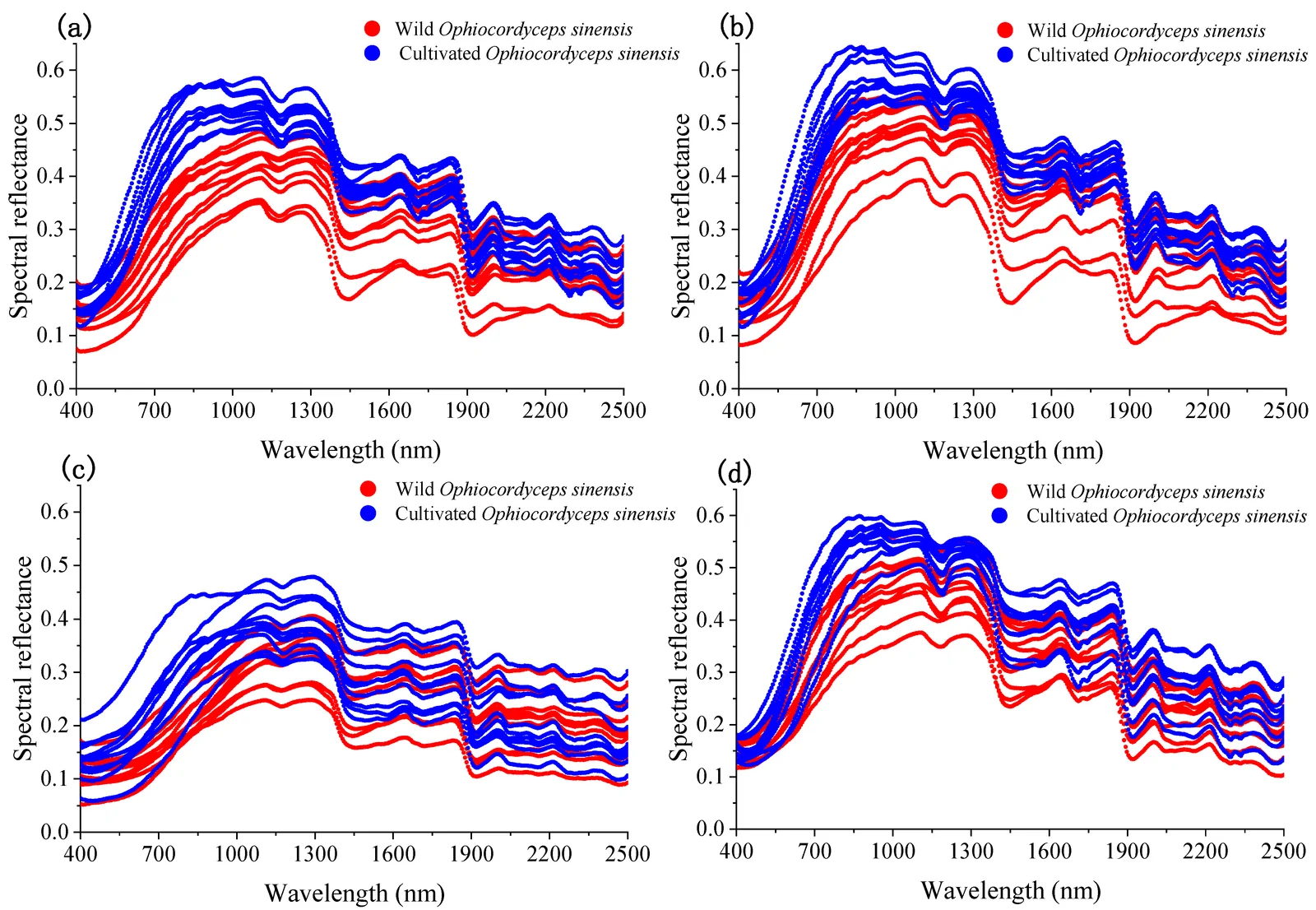
Hyperspectral reflectance curves of 20 *Ophiocordyceps sinensis* samples (10 wild and 10 cultivated). Among these, (**a**), (**b**) and (**c**) were derived from different parts of the same intact *Ophiocordyceps sinensis*, which were the spectral reflectance curves of the intact part, the larva part and the stroma part, respectively, while (**d**) is the spectral reflectance curve of the real stroma-missing *Ophiocordyceps sinensis*.

By cyclically processing the VNIR and SWIR hyperspectral images of each *Ophiocordyceps sinensis* sample, the hyperspectral reflectance curve of each sample could be obtained. The VNIR (400–1000 nm) and SWIR (930–2500 nm) spectral ranges overlap in the 930–1000 nm region. As the VNIR and SWIR cameras provide 108 and 288 spectral bands, respectively, the spectral sampling across the 400–2500 nm range is relatively sparse. Consequently, the noise effects at the spectral boundaries of the two cameras were not pronounced and were not expected to substantially affect the overall spectral characteristics. Within the overlapping region of 930–1000 nm, however, the SWIR data exhibited higher noise levels than the VNIR data. Therefore, to minimize the influence of the relatively high noise in the SWIR overlap region, the SWIR data from 930–1000 nm were excluded. The VNIR data from 400–1000 nm were then concatenated with the SWIR data from 1003–2500 nm (i.e., 1003 nm represents the first retained SWIR wavelength after removal of the 930–1000 nm overlap region), with the spectra joined at approximately 1000 nm to form a continuous spectrum covering 400–2500 nm. No additional removal or truncation of the spectral boundary bands was performed, and the retained spectral data were used as model inputs. Figure 4 shows the hyperspectral reflectance curves of some wild and cultivated *Ophiocordyceps sinensis*.

#### 2.2.3. Dataset Construction Method

Each of the 917 intact *Ophiocordyceps sinensis* samples was divided into two parts: the larva and the stroma, and the corresponding single-part reflectance curves were obtained separately. The spectral reflectance curves of the intact part, larva part, and stroma part of each intact *Ophiocordyceps sinensis* sample were all collected and constructed into DS_IntP, DS_LarP, and DS_StrP, respectively. Then, the three datasets (DS_IntP, DS_LarP, and DS_StrP) were mixed into DS_MixILSP, and the two datasets (DS_IntP and DS_LarP) were mixed into DS_MixILP. In addition, the spectral reflectance curves of real stroma-missing *Ophiocordyceps sinensis* samples were collected to construct DS_MisStrP.

DS_IntP, DS_LarP, and DS_StrP originate from the same batch of *Ophiocordyceps sinensis* samples, thus possessing consistent sample numbers and categories, corresponding only to different parts. When constructing the mixed datasets, the data of the three parts were encoded first; the intact part, larva part, and stroma part were designated as 0, 1, and 2, respectively. Subsequently, a part encoding table was established using the sample number as the index, and a part code was randomly generated for each sample. Based on this code, the spectral data of the corresponding sample were extracted from the three homologous datasets, and the extracted data were recombined to form a new mixed dataset. Among these, the data of DS_MixILSP were randomly selected from the three datasets of the intact part, larva part, and stroma part; DS_MixILP were randomly selected only from the two datasets of the intact part and larva part.

To reduce the randomness, 10 datasets of random part encoding tables were independently generated for DS_MixILSP and DS_MixILP, and 10 mixed datasets were constructed accordingly; subsequent experiments were conducted independently on the 10 datasets, and the mean accuracy of the ten datasets of experiments was used to evaluate the model performance. The quantity of each part in the DS_MixILSP and DS_MixILP during the ten repeated experiments is shown in Table 3.

### 2.3. Methods

Considering the characteristics of the dataset, the nature of the classification task, and commonly adopted modeling approaches in the field, five representative machine learning algorithms were selected for comparative evaluation. In addition, a FCNN was included as a deep learning model. Collectively, these models encompass distinct modeling paradigms, including linear classification, kernel-based learning, Bagging-based ensemble learning, Boosting-based ensemble learning, and deep learning, thereby enabling a systematic comparison of their classification performance and generalization capability within a unified experimental framework. Ultimately, the optimal model will be selected based on identification accuracy as the standard method for the instrument to identify wild and cultivated *Ophiocordyceps sinensis*.

#### 2.3.1. Machine Learning Methods

This study selected five ML models: LR, SVM, RF, XGBoost and LightGBM, for identifying wild and cultivated *Ophiocordyceps sinensis*. These models demonstrate good representativeness in high-dimensional feature processing capability, generalization performance, and engineering application maturity.

LR was employed as a linear classification baseline to assess the linear separability of the data; SVM was used to model potential nonlinear classification relationships; RF, a representative Bagging-based ensemble method, was adopted to achieve robust classification through the aggregation of multiple decision trees. XGBoost and LightGBM represent Boosting-based ensemble methods and were used to capture complex nonlinear relationships and potential interactions among features.

By adopting a unified data preprocessing and evaluation system for the comparative analysis of multiple models, biases caused by single model selection can be avoided, thereby more objectively reflecting the impact of the optimal detection of part selection on identification performance.

#### 2.3.2. Deep Learning Methods

FCNN, also widely referred to as the multilayer perceptron (MLP), constitutes one of the most fundamental architectures in deep learning. Its canonical structure comprises an input layer, a succession of hidden layers, and an output layer, with each neuron in a given layer establishing dense, all-to-all connections to every neuron in the subsequent layer. By incorporating nonlinear activation functions, the network gains the capacity to approximate arbitrary continuous functions. In the training phase, the backpropagation algorithm, grounded in the chain rule of calculus, is employed to efficiently compute the gradients of a predefined loss function with respect to all trainable parameters. These gradients are subsequently utilized by gradient-based optimizers, most notably stochastic gradient descent and its adaptive variants, to iteratively update the parameters, progressively minimizing the empirical risk.

In this study, an FCNN model was constructed, comprising a specific architecture with six hidden layers and one output layer. The neuron configuration for the hidden layers was set to (512, 256, 128, 64, 32, 16). To mitigate the vanishing gradient problem and accelerate convergence, the rectified linear unit (ReLU) activation and batch normalization (BN) are applied after each fully connected operation. Furthermore, a batch normalization (BN) layer is introduced following each fully connected operation. Given the substantial number of model parameters, L2 regularization and dropout techniques are incorporated into the hidden layers to enhance generalization ability and prevent overfitting. The output layer consists of two neurons and utilizes the Softmax function to output the classification probabilities for wild and cultivated *Ophiocordyceps sinensis*.

#### 2.3.3. Validation

The hyperspectral identification system for wild and cultivated *Ophiocordyceps sinensis* was developed based on the Python 3.11, utilizing scikit-learn version 1.7.1 to construct the ML models.

All ML models were trained and evaluated using a nested ten-fold stratified cross-validation strategy to ensure unbiased performance estimation. The dataset was divided into ten stratified folds using a fixed random seed of 42, with each fold serving as the external validation set while the remaining nine folds constituted the training set. To prevent data leakage, sample-wise standard normal variate (SNV) preprocessing was fitted exclusively on the training set of each outer fold; the calculated parameters (mean and standard deviation) were then applied to both the training and validation sets. For hyperparameter tuning, an inner five-fold stratified grid search was performed on the training set within each outer fold (also using seed 42 for consistency), with search ranges detailed in Table 4. The optimal hyperparameters were selected based on the macro F1 score to account for class imbalance. A unified class-weighting strategy was employed across all models: LR, SVM, and RF utilized the class_weight = ‘balanced’ argument, while LightGBM and XGBoost incorporated equivalent inverse class frequency weights via sample-weight parameters. The final reported performance metrics represent the mean and standard deviation calculated across the ten outer validation folds. For the independent testing procedure, a five-fold grid search was performed on the complete training dataset to identify the optimal hyperparameters, we then trained and saved the model on the entire training set, and finally evaluated it on the independent test dataset. Detailed hyperparameter information is shown in Table 4.

The FCNN model was trained using ten-fold cross-validation to ensure robust performance evaluation. The model parameters were optimized via the Adam optimizer, whose adaptive learning rate mechanism proves particularly effective in handling sparse gradients. The initial learning rate was set to 0.001. We adopted the cross-entropy loss function to quantify the divergence between the predicted probability distribution and the ground-truth labels. Considering the inherent characteristics of the hyperspectral dataset, a batch size of 16 was chosen to introduce adequate stochasticity during optimization. The model was trained for a total of 1000 epochs.

To evaluate the classification performance under the imbalanced class distribution, the use of overall accuracy alone could be insufficient. In addition to overall accuracy (Acc) and mean accuracy (mAcc), class-specific performance metrics were introduced to assess the model’s performance on both classes. These metrics include precision (Pre), recall (Rec), specificity (Spe), and F1 score (F1), with the calculation as follows:

(3) $$Acc=\frac{TP+TN}{TP+TN+FP+FN}$$

(4) $$mAcc=\left(\frac{1}{N}\right)\sum_{K=1}^{N}{Acc}_{K}$$

(5) $$Pre=\frac{TP}{TP+FP}$$

(6) $$Rec=\frac{TP}{TP+FN}$$

(7) $$Spe=\frac{TN}{TN+FP}$$

(8) $$F1=2\times \frac{Pre\times Rec}{Pre+Rec}$$

where *TP* is true positives (i.e., wild samples correctly predicted as wild); *TN* is true negatives (i.e., cultivated samples correctly predicted as cultivated); *FP* is false positives (i.e., cultivated samples incorrectly predicted as wild); *FN* is false negatives (i.e., wild samples incorrectly predicted as cultivated). *N* is the number of independent experiments and ${Acc}_{K}$ is the accuracy of the k-th experiment.

## 3. Results

Based on the simulated datasets constructed from intact *Ophiocordyceps sinensis* and the dataset of real stroma-missing *Ophiocordyceps sinensis*, single-part identification, mixed-part identification, and the real scene testing experiments were conducted to compare the classification performance of ML models under different detecting parts and different training strategies. This study utilized six models: LR, SVM, RF, XGBoost, LightGBM and FCNN for comparative analysis. The experimental results of each stage are explained below.

### 3.1. Single Part Identification Results

To evaluate the spectroscopic identification information contribution of different parts of *Ophiocordyceps sinensis* to the identification of wild and cultivated categories, DS_IntP, DS_LarP, and DS_StrP were utilized to train the six models, and ten-fold cross-validation was adopted to evaluate model performance. The Acc results are shown in Table 5, and the complete performance indicators are listed in Table A1 of Appendix A.

As can be seen from Table 5, all models achieved the highest identification accuracy on DS_IntP. Among these, the FCNN, LR, and SVM models all achieved a mean accuracy of 100.00% on DS_IntP, demonstrating extremely high identification capability. When only DS_LarP was used for modeling, the LR and SVM models maintained 100.00% accuracy, while the FCNN model achieved 99.89%, indicating that the larva part contains rich identification information.

In contrast, when only DS_StrP was used to build the models, the accuracy of the LR and SVM models dropped to 98.91% and 98.69% on DS_StrP, respectively, and FCNN dropped to 98.15%. For tree-based models, this downward trend was more pronounced. The accuracies of the RF model on DS_IntP, DS_LarP, and DS_StrP were 98.14%, 97.71%, and 95.09%, respectively; the XGBoost model was 98.58%, 97.60%, and 94.88%, respectively; and the LightGBM model was 98.36%, 97.93%, and 95.53%, respectively.

Overall, the six models exhibited the consistent pattern: the intact part dataset achieved the highest identification accuracy, followed by the larva part, and the stroma part was the lowest. The spectroscopic identification information contribution of the larva part is greater than that of the stroma part; meanwhile, the intact part contains the most abundant identification information, and the larva part could retain most of the key identification features, whereas relying solely on the stroma part leads to a significant decline in identification performance.

### 3.2. Mixed Part Identification Results

To simulate the scene where different parts randomly appear in practical application processes, two types of mixed datasets (DS_MixILSP, DS_MixILP) were further constructed to simulate the existence of broken *Ophiocordyceps sinensis* in the real market. Ten experiments were conducted using 10 datasets of DS_MixILSP and DS_MixILP, and each experiment utilized ten-fold cross-validation to calculate model accuracy. After ten experiments, the mean of the ten accuracies was calculated to evaluate the model performance under that dataset. The Acc results are shown in Table 6 and Table 7, respectively. And the complete performance indicators are respectively listed in Table A2 and Table A3 of Appendix A.

As shown in Table 6, on the 10 repeated experiments of DS_MixILSP, the mean accuracy of the SVM model was 98.77%, LR was 98.75%, and FCNN was 98.05%. The mean accuracies of the RF, XGBoost and LightGBM models were 94.56%, 94.95%, and 95.07%, respectively.

As shown in Table 7, on DS_MixILP, the mean accuracy of all models was higher than that on DS_MixILSP. Among them, the mean accuracy of FCNN, LR, and SVM reached 99.79%, 99.60%, and 99.52%, respectively; the mean accuracies of the RF, XGBoost, and LightGBM models were 96.60%, 96.59%, and 97.33%, respectively.

Further comparing the two mixed scenes revealed that when the randomly mixed data contains stroma part data, model accuracy is generally lower; conversely, when only the intact part and larva part are retained, the performance of all models is improved. Among these, the accuracy of the LR model increased from 98.75% to 99.60%, SVM increased from 98.77% to 99.52%, and FCNN increased from 98.05% to 99.79%. This indicates that the stroma part, as a separate input, pulls down the identification capability of the models. In contrast, the larva part, as a separate input, can provide a spectroscopic identification information contribution similar to that of the intact part.

### 3.3. Real-Scene Testing

To verify the applicability of the models in the real market scene, DS_MisStrP was used to test the single-part models and mixed-part models established previously. As the identification results of the RF, XGBoost and LightGBM models were not ideal in the above experiments, the three top-performing architectures, the FCNN, LR, and SVM models, were used for testing in this section. All sample data from DS_IntP, DS_LarP, DS_StrP, DS_MixILSP, and DS_MixILP were utilized to train and save the trained models. The respective trained models were named Mod_IntP_FCNN, Mod_LarP_FCNN, Mod_StrP_FCNN, Mod_MixILSP_FCNN, and Mod_MixILP_FCNN, Mod_IntP_LR, Mod_LarP_LR, Mod_StrP_LR, Mod_MixILSP_LR, and Mod_MixILP_LR, as well as Mod_IntP_SVM, Mod_LarP_SVM, Mod_StrP_SVM, Mod_MixILSP_SVM, and Mod_MixILP_SVM. The test results of the single-part models are shown in Table 8.

As shown in Table 8, the Mod_IntP_FCNN, Mod_IntP_LR, and Mod_IntP_SVM achieved the highest classification accuracy on real stroma-missing samples, reaching 98.52%, 98.52%, and 97.78%, respectively. For the models trained using DS_LarP, the accuracy was 98.52%, 97.04%, and 97.04%, respectively; the models trained using DS_StrP exhibited the lowest performance, with FCNN, SVM, and LR achieving accuracies of 90.37%, 88.15%, and 87.41%, respectively.

Subsequently, the models trained with mixed-part datasets were tested. Because the mean accuracy of DS_MixILSP in the previous experiment was lower than that of DS_MixILP, the results of the two mixed training strategies on real samples were further compared. The experimental results are shown in Table 9 and the complete performance indicators are listed in Table A4 and Table A5 of Appendix A.

As shown in Table 9, the mean accuracies of the models trained based on DS_MixILSP over 10 independent experiments were 96.45% (SVM), 96.30% (FCNN), and 95.63% (LR), respectively; while the mean accuracies of the models trained on DS_MixILP reached 97.04% (LR), 96.97% (FCNN), and 96.81% (SVM), respectively.

**Figure 5:**
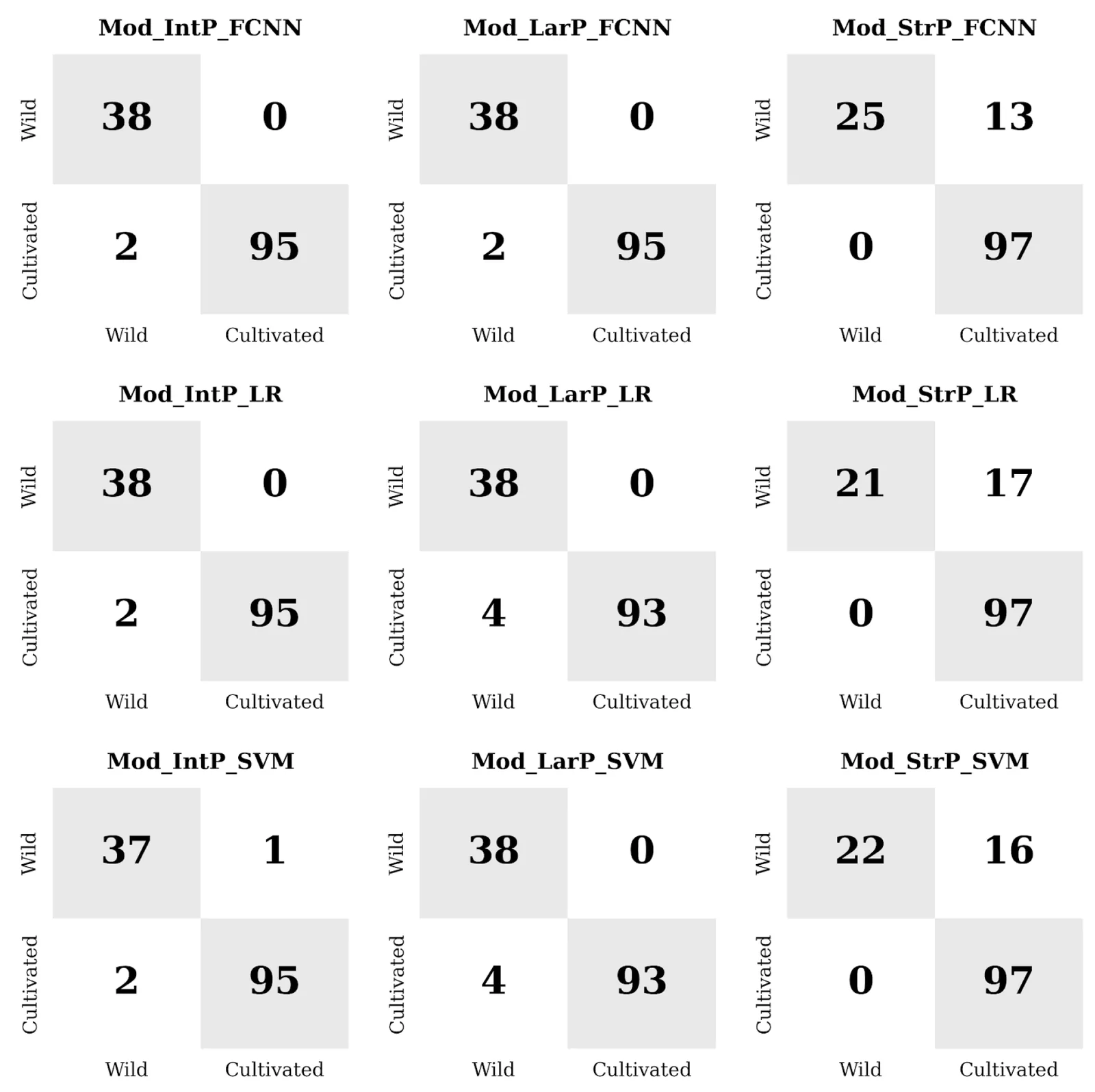
Confusion matrices for models trained on the single-part datasets when tested on DS_MisStrP.

**Figure A1:**
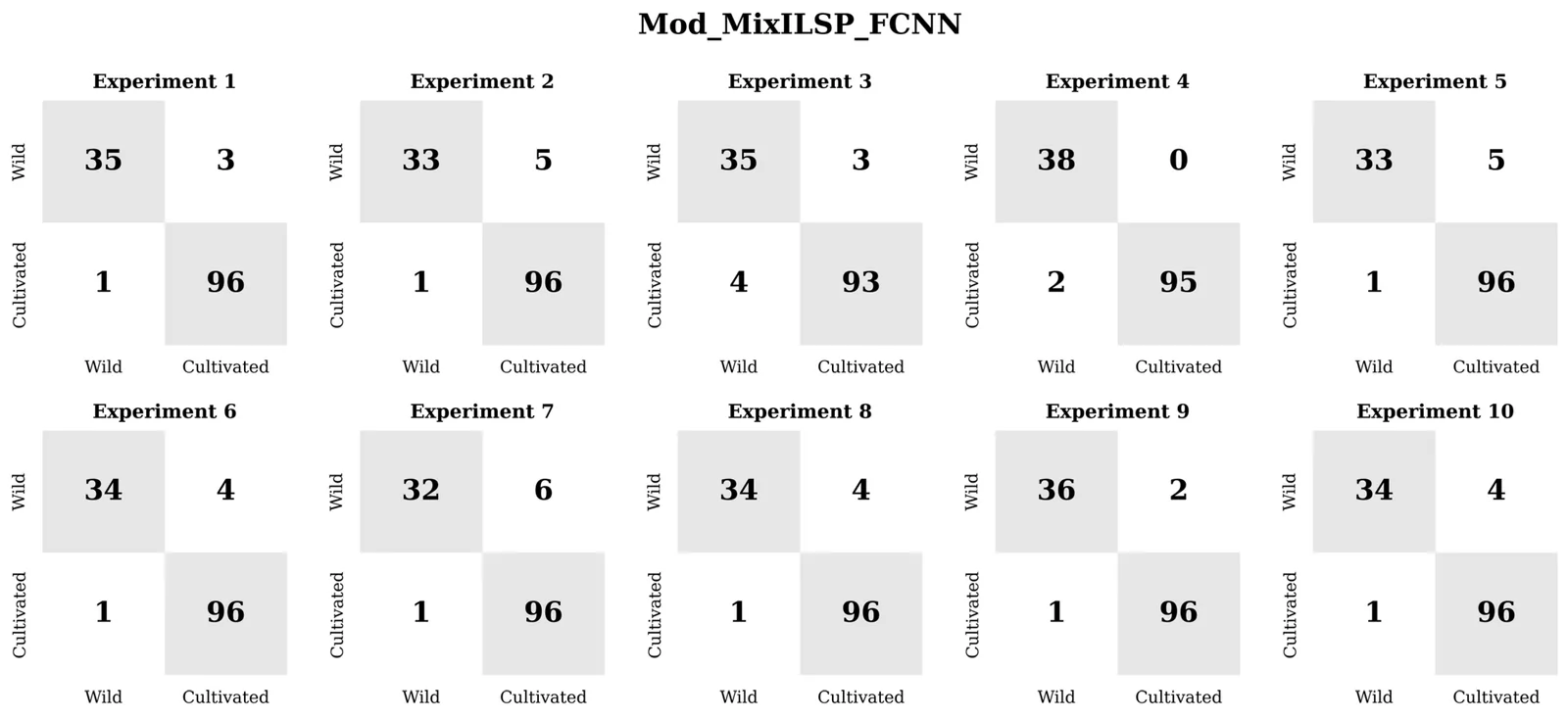
Confusion matrices of FCNN trained on the mixed-part datasets (DS_MixILSP) when tested on DS_MisStrP.

**Figure A2:**
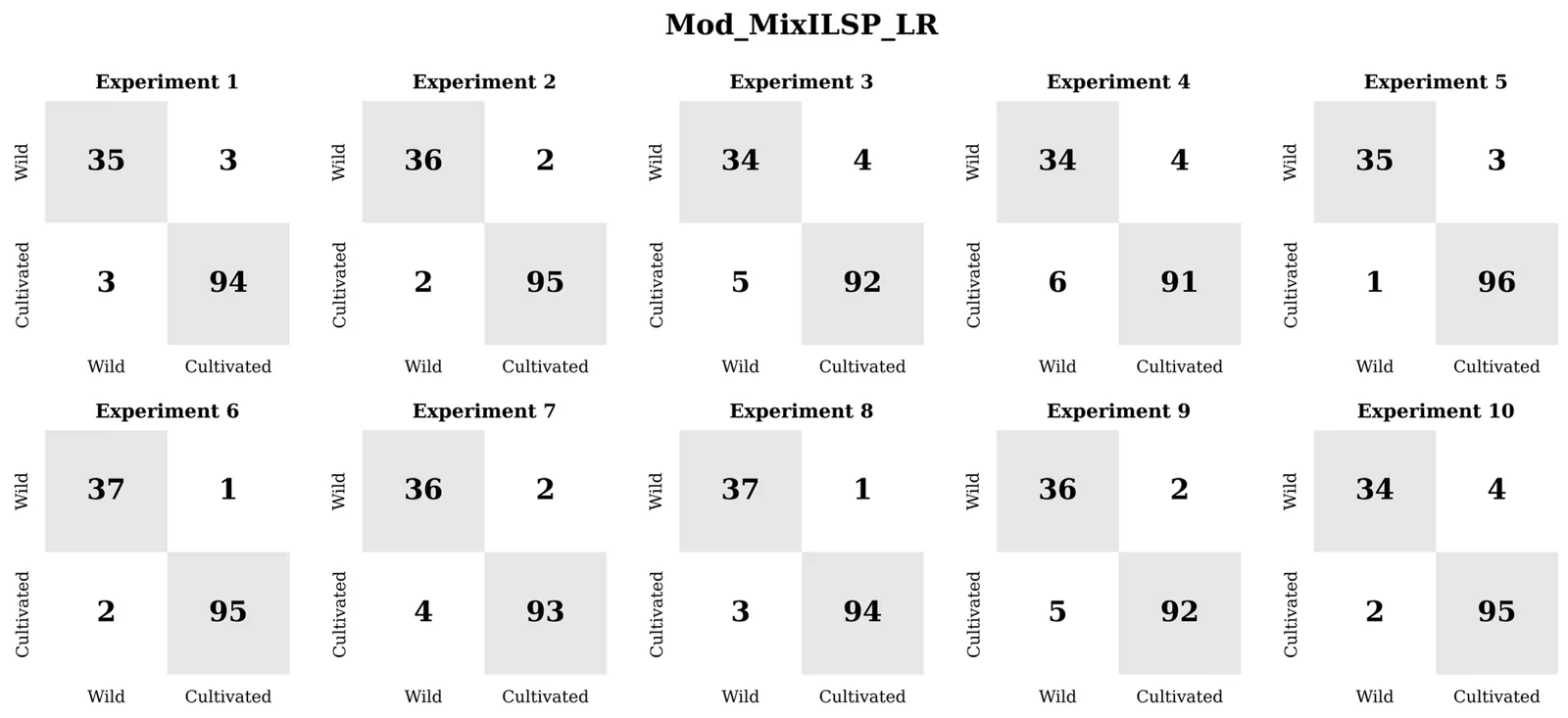
Confusion matrices of LR trained on the mixed-part datasets (DS_MixILSP) when tested on DS_MisStrP.

**Figure A3:**
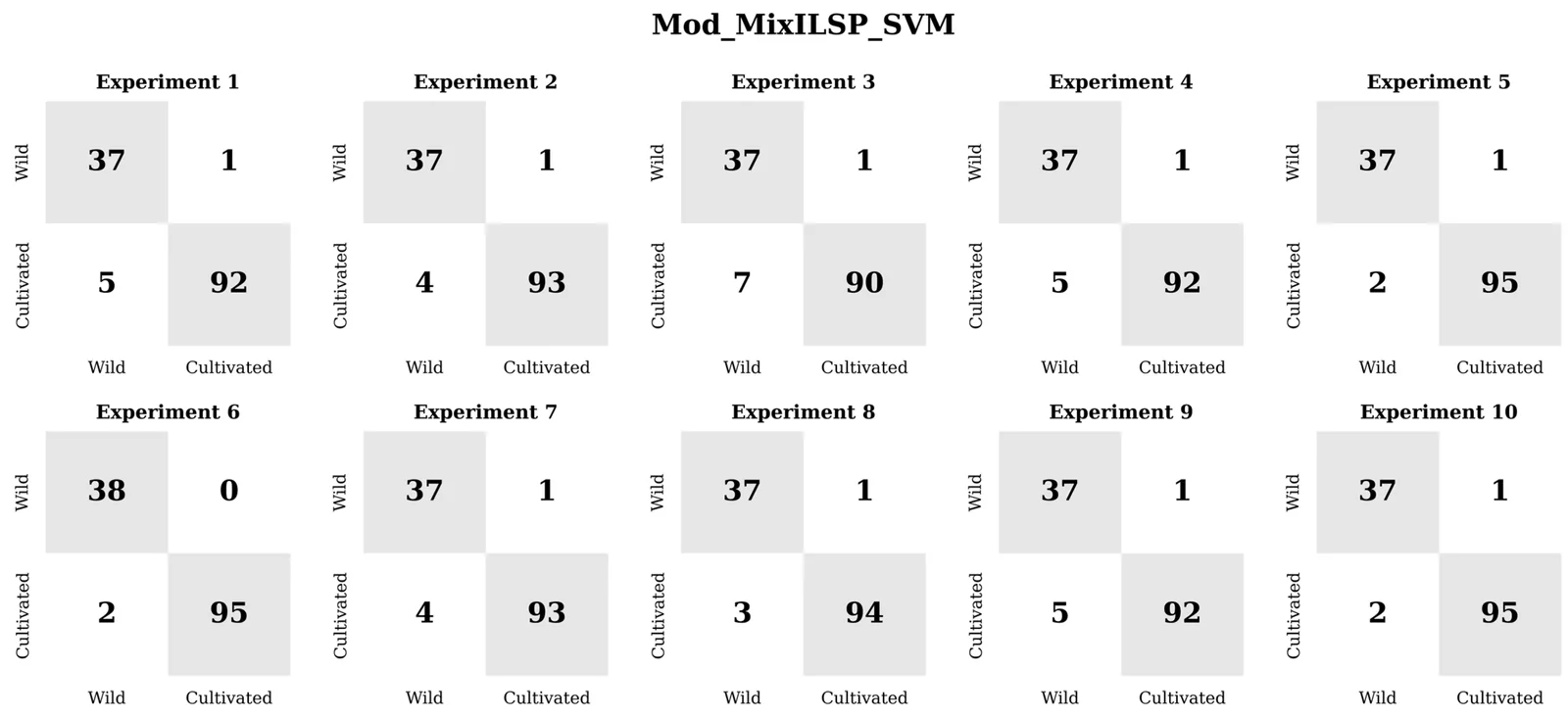
Confusion matrices of SVM trained on the mixed-part datasets (DS_MixILSP) when tested on DS_MisStrP.

**Figure A4:**
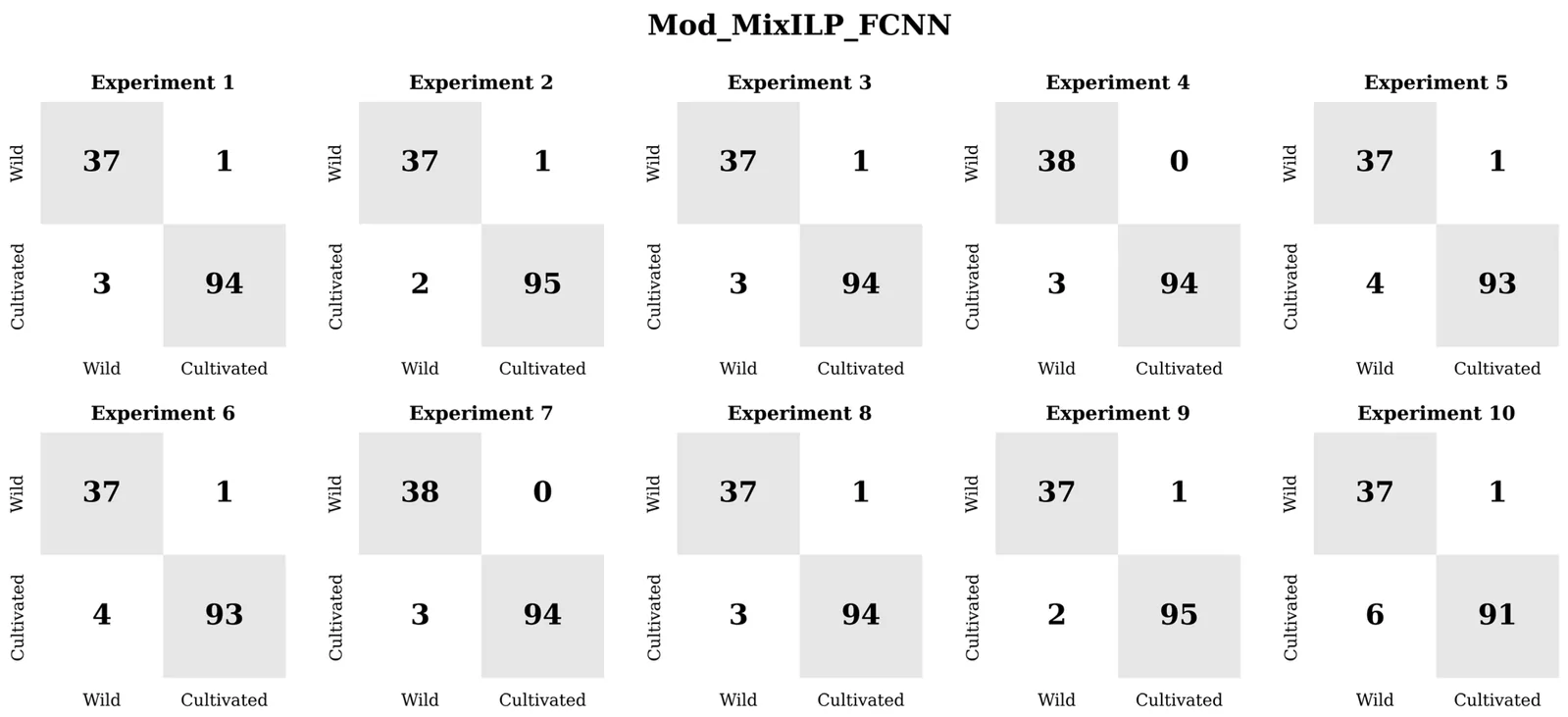
Confusion matrices of FCNN trained on the mixed-part datasets (DS_MixILP) when tested on DS_MisStrP.

**Figure A5:**
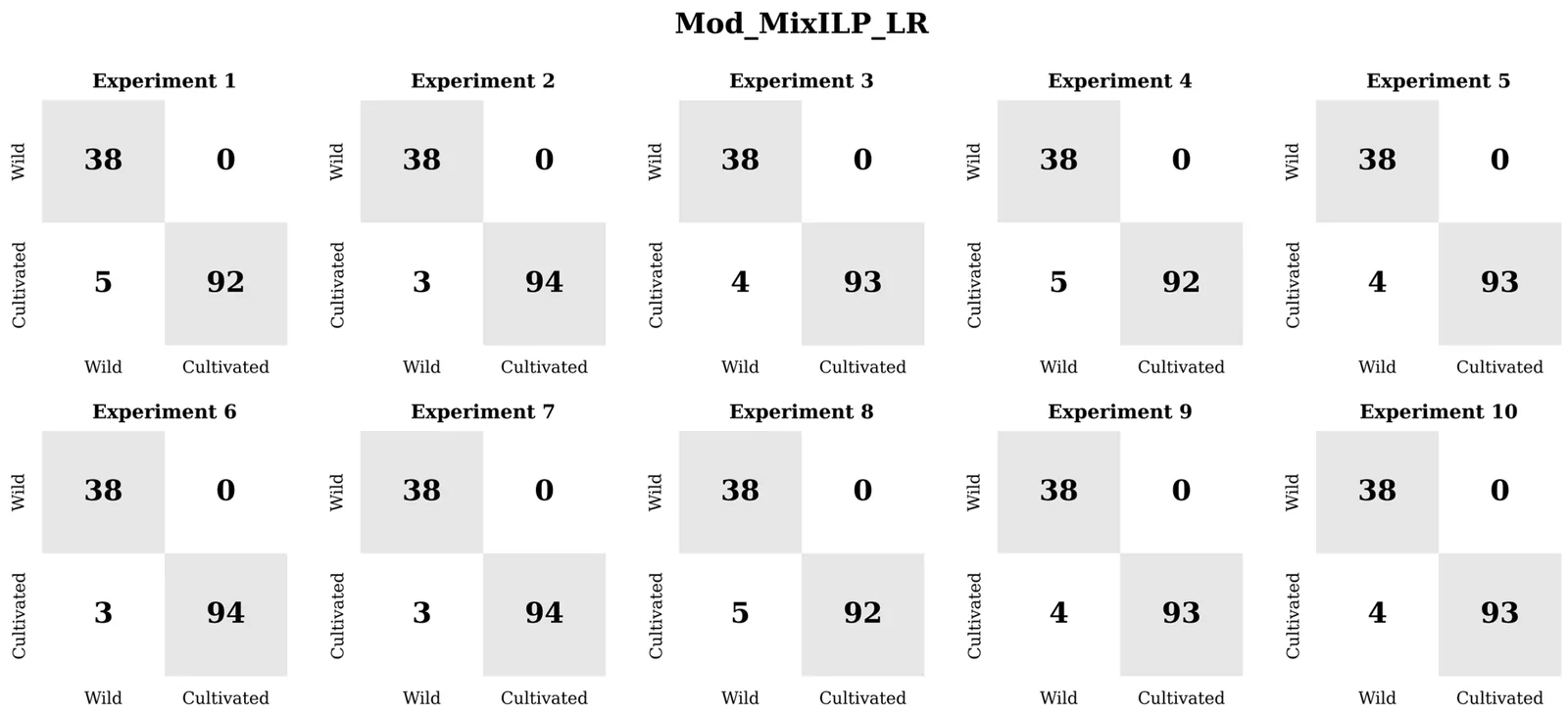
Confusion matrices of LR trained on the mixed-part datasets (DS_MixILP) when tested on DS_MisStrP.

**Figure A6:**
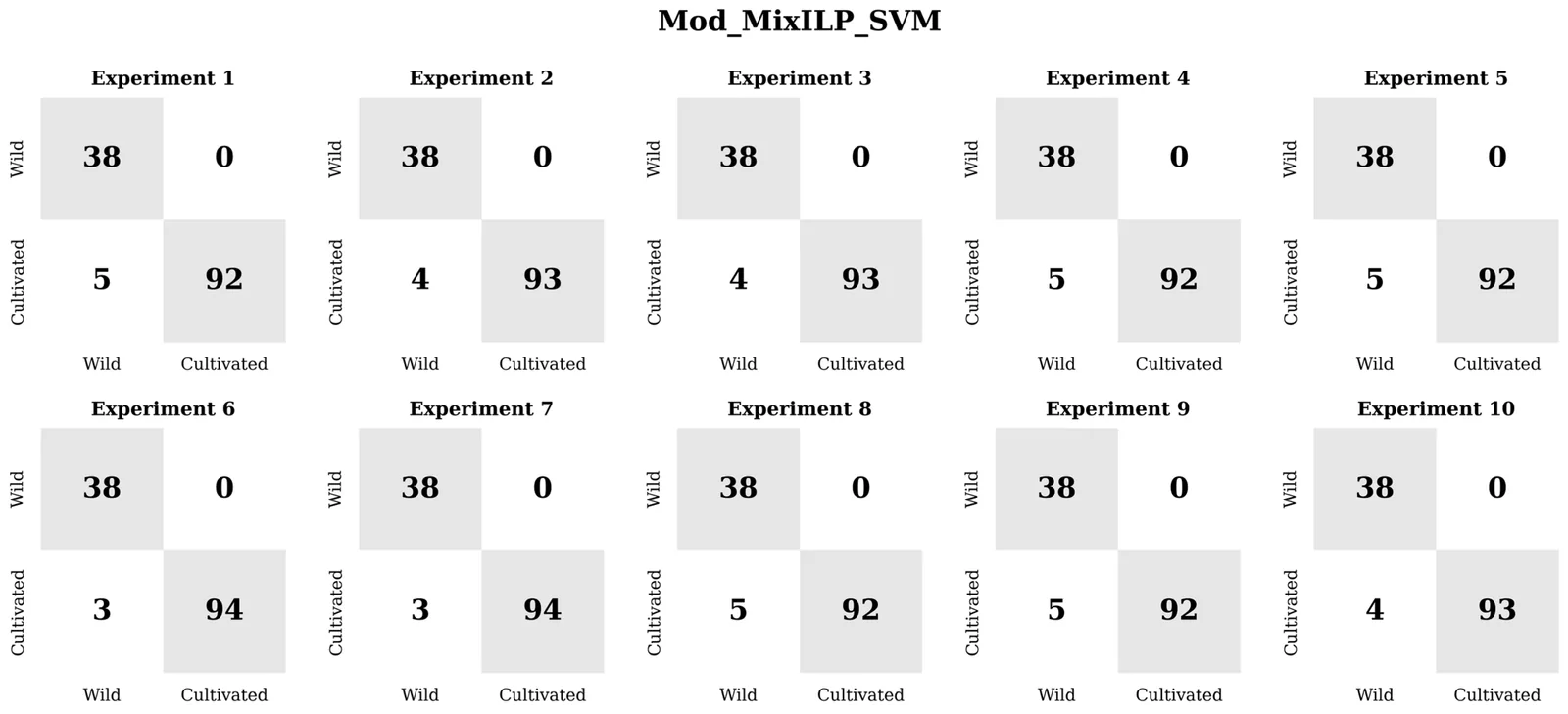
Confusion matrices of SVM trained on the mixed-part datasets (DS_MixILP) when tested on DS_MisStrP.

Figure 5 is the confusion matrix of the single-part datasets, and the confusion matrices of the mixed-part datasets are placed in Figure A1, Figure A2, Figure A3, Figure A4, Figure A5 and Figure A6 of Appendix A. The confusion matrix results from the independent real test dataset clearly show the misclassification patterns of different models. Models trained on the intact part and the larva part successfully recognized all or nearly all wild samples with only a very small number of cultivated samples misclassified as wild. In contrast, models trained on the stroma part misidentified a large proportion of wild samples as cultivated. Furthermore, in the mixed-part dataset experiments, the models trained on intact and larva mixtures maintained stable and highly accurate predictions for wild samples across all repetitions, whereas adding the stroma part to the training data led to noticeable fluctuations and increased errors on wild samples. These findings confirm that the larva part preserves the essential discriminative features of intact samples, while the stroma part introduces interference when identifying stroma-missing samples.

Judging from the results of the 10 independent experiments, between DS_MixILSP and DS_MixILP, the models trained on DS_MixILP maintained better stability, with smaller accuracy fluctuations across experiments, whereas the accuracy of models trained on DS_MixILSP fluctuated more by comparison. Furthermore, the mean accuracy of models trained on DS_MixILP on real stroma-missing samples was higher than that of DS_MixILSP.

## 4. Discussion

### 4.1. Analysis of Contribution Differences Among Different Parts

For *Ophiocordyceps sinensis*, morphological differences exist among the larva and stroma parts; such differences may be reflected in hyperspectral information, thereby affecting hyperspectral identification performance. In engineering applications, it is necessary to clarify which part should be selected to acquire hyperspectral data based on the differences among various parts.

**Figure 6:**
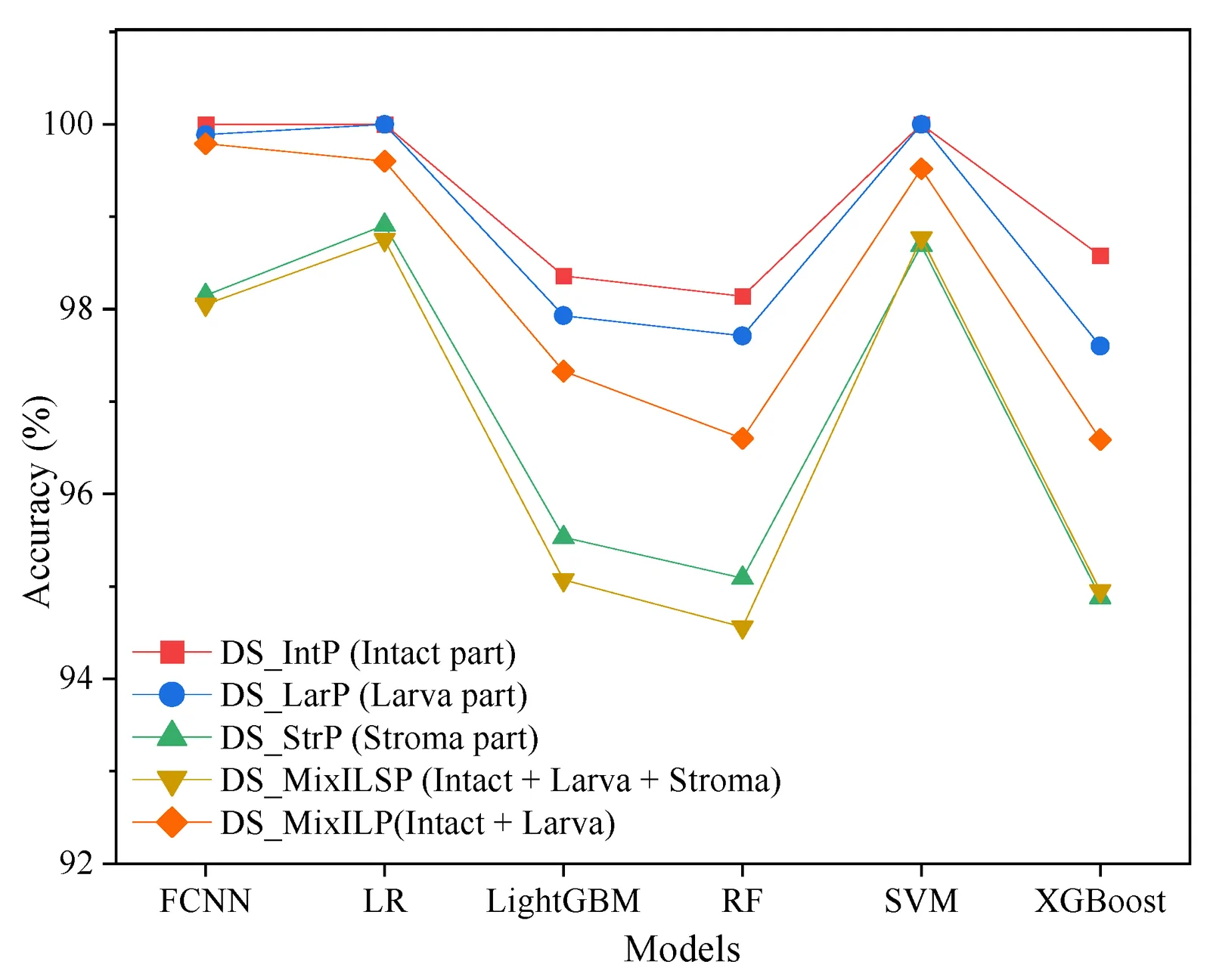
Comparison of the accuracies of single-part datasets (DS_IntP, DS_LarP and DS_StrP) and mixed-part datasets (DS_MixILSP, DS_MixILP) using the six models.

Through single-part identification experiments and mixed-part identification experiments, the spectroscopic identification information contribution differences of different parts to the identification of wild and cultivated *Ophiocordyceps sinensis* were analyzed. The accuracies of the single part datasets (DS_IntP, DS_LarP, and DS_StrP) and mixed part datasets (DS_MixILSP, DS_MixILP) using the six models were compared together, as shown in Figure 6.

First, it can be seen from the figure that the performance of the six models exhibits a consistent pattern: the performance of the LR, SVM and FCNN models shows advantages compared with other models. The strong performance of LR, SVM, and FCNN suggests that the classification boundary may be relatively simple in the standardized spectral feature space. Conversely, RF, XGBoost and LightGBM capture complex feature interactions by ensembling multiple decision trees, which may instead amplify noise interference and reduce identification performance.

Second, comparing the accuracies of the five datasets on each model also reveals a consistent pattern, namely, the accuracy of the intact part dataset is the highest, followed by the larva part, and the stroma part is the lowest. Among these, the accuracies of the LR, SVM, and FCNN models on the intact part all reached 100.00%, maintained high accuracies of 100.00% (LR, SVM) and 99.89% (FCNN) on the larva part, while dropping to 98.91% (LR), 98.69% (SVM), and 98.15% (FCNN) on the stroma part. This indicates that differences exist in the identification information carried by the larva part and the stroma part of *Ophiocordyceps sinensis*. The intact part simultaneously contains the part information of both the larva and the stroma, and therefore can retain the spectral differences between wild and cultivated *Ophiocordyceps sinensis* to the maximum extent.

In addition, as shown in the figure, some models achieved 100% accuracy. Although these results indicate excellent class separability under the current experimental conditions, they should be interpreted with caution. The datasets were constructed from intact *Ophiocordyceps sinensis* samples, with stroma-missing samples excluded. Therefore, the samples within each dataset were relatively consistent in morphology, which may have reduced within-class spectral variability and resulted in performance that was somewhat better than might be expected under real scene. In addition, the sample size in this study was relatively limited and the wild *Ophiocordyceps sinensis* samples were collected from Xizang and did not cover other production regions such as Qinghai. Increasing the sample size and expanding the geographic coverage may introduce greater sample variability and consequently lead to a slight decrease in accuracy. Therefore, these results should be interpreted in the context of the specific datasets, with greater emphasis placed on the comparative performance and consistency of the models across different datasets.

**Figure 7:**
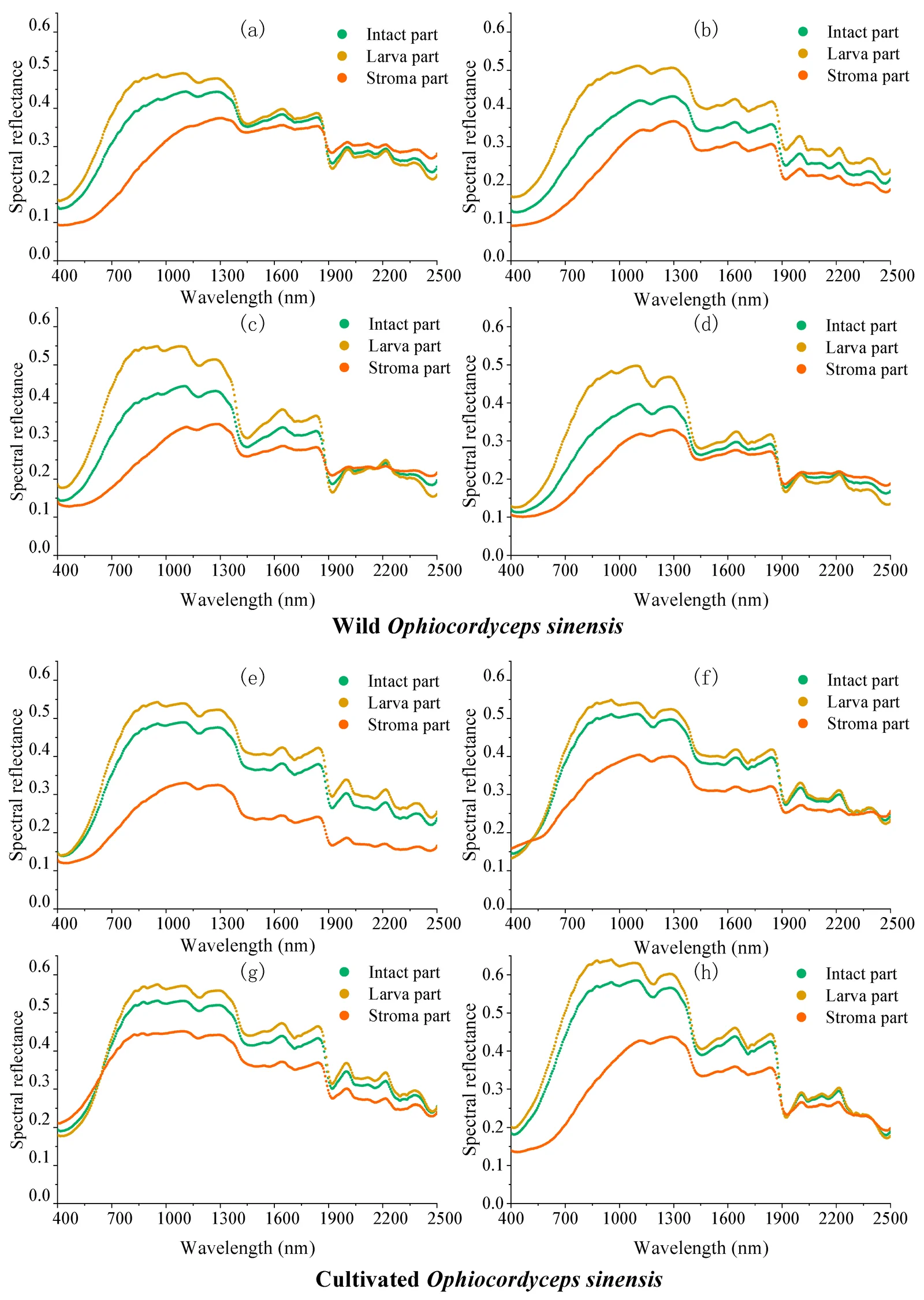
Comparison of the spectral reflectance curves of three different parts of eight *Ophiocordyceps sinensis* samples, where (**a**–**d**) are wild *Ophiocordyceps sinensis* and (**e**–**h**) are cultivated *Ophiocordyceps sinensis*.

Figure 7 shows the comparison of the spectral reflectance curves of three different parts of eight *Ophiocordyceps sinensis* samples, demonstrating the signal-to-noise ratio difference caused by brightness. Although the larva part and the stroma part originate from the same organism, differences exist between them in tissue structure, growth mode, and chemical composition. Hyperspectral technology reflects the comprehensive response of differences in sample surface chemical composition and tissue structure [28]; the larva part is the primary accumulation area of active components and the stroma is the fungal fruiting body [29], thus the reflectance curves of different parts possess relatively obvious differences, thereby generating different identification capabilities. The experimental results demonstrate that the intact part can simultaneously retain the feature information provided by the larva and the stroma parts, and therefore exhibits optimal identification performance.

Additionally, when the mixed dataset only contained data of the intact part and the larva part, the overall model performance declined slightly. Further comparing the results of DS_MixILSP and DS_MixILP reveals that when the stroma part was introduced into the randomly mixed data, all models experienced varying degrees of accuracy decline. When only the larva part was retained as an independent input, although the model accuracy declined slightly, the overall level remained relatively high; however, when the stroma part was introduced as an independent input, the model performance was even inferior to the model performance of the DS_StrP. This indicates that the information contained in the stroma part has large differences from the larva part, thereby exerting a certain impact on the classification models. In contrast, the larva part, as a separate input, can provide a spectroscopic identification information contribution similar to that of the intact part. Therefore, it is recommended not to incorporate stroma part data into mixed part datasets.

### 4.2. Hyperspectral Identification Capability for Broken Ophiocordyceps sinensis

Differences exist among different parts of *Ophiocordyceps sinensis*, but how much does this difference affect the accuracy of identifying stroma-missing *Ophiocordyceps sinensis*? By uniformly testing on the independent dataset of stroma-missing samples, the difference that different parts have on hyperspectral identification was quantitatively compared. Simultaneously, such testing answers whether specialized hyperspectral detection instruments possess high-accuracy detection capabilities for stroma-missing *Ophiocordyceps sinensis* widely present in the real market scene.

**Figure 8:**
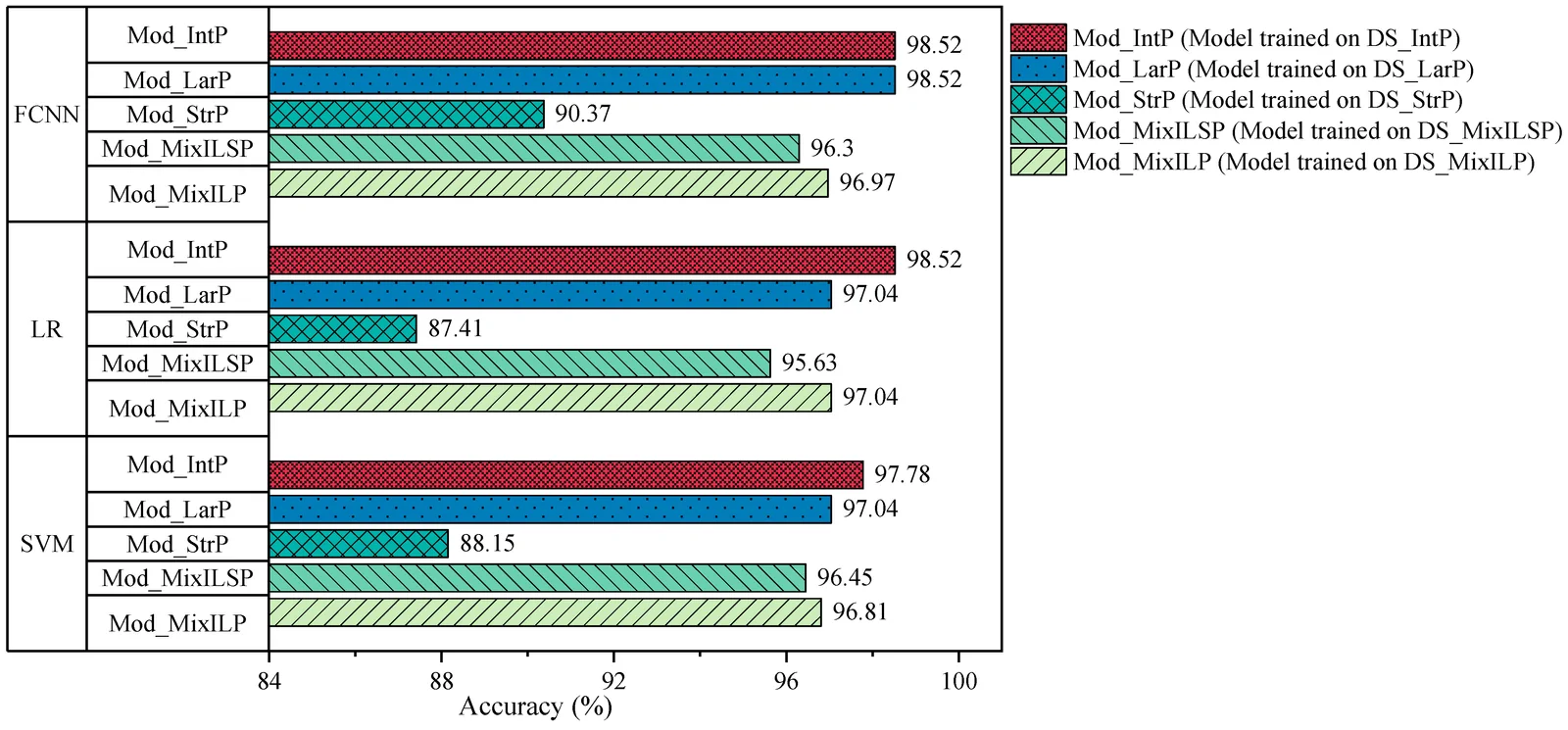
The comparison of the accuracies of each model on the test dataset.

Independent dataset testing was conducted using 135 real stroma-missing *Ophiocordyceps sinensis* circulating in the market, and the test objects were the models trained on single part and mixed part datasets (Mod_IntP, Mod_LarP, Mod_StrP, Mod_MixILSP, and Mod_MixILP). By comparing the accuracies of the models on the independent test dataset, the magnitude of the spectroscopic identification information contribution of different parts to wild and cultivated identification was quantitatively given. Figure 8 is a comparison chart of the accuracies of each model on the test dataset.

As can be seen from the figure, for the LR models, the accuracy differences between the intact part dataset and the larva part dataset as well as the stroma part dataset on real stroma-missing samples were 1.48% and 11.11%, respectively; for SVM models, the differences were 0.74% and 9.63%, respectively; and for FCNN models, the differences were 0 and 8.15%, respectively. These quantitative results demonstrate that the difference between the intact part and larva part is minimal, whereas the difference with the stroma part is large.

The test results of Mod_StrP are very poor, because the stroma and the larva parts are two different parts of *Ophiocordyceps sinensis*, and there are significant differences in morphology and biological components between the two parts. The test results show that the spectral response signals of the two parts also reflect this difference, so it is not feasible to use the data of one part to identify the category of another part data.

The test results of Mod_LarP are not as good as those of Mod_IntP. The data in DS_LarP and DS_MisStrP are consistent in sample morphology, and both contain only the larva part; but the data in DS_IntP also synthesize the stroma part. This situation can be explained by the stroma-missing samples in Figure 1: From the samples (d–f), it can be seen that the stroma-missing samples are different in morphology, some completely missing the stroma part, and some still retain a little, which leads to insufficient representation of the simulation dataset. This explanation can be confirmed from the Mod_MixILP test accuracy between the Mod_IntP and the Mod_LarP.

In the *Ophiocordyceps sinensis* identification task, models trained on intact samples already possess strong adaptive capabilities and can be applied to the detection of common stroma-missing samples in the market, thereby reducing the difficulty of sample screening and standardized processing during actual application.

Comprehensively combining the results provides a basis for the optimal detecting part of the *Ophiocordyceps sinensis* hyperspectral identification instrument. When the instrument design pursues both the highest identification accuracy and a broad scope for engineering application, the intact part of wild *Ophiocordyceps sinensis* should be collected for model training. The trained model is applicable to intact *Ophiocordyceps sinensis* and stroma-missing *Ophiocordyceps sinensis*, and can be identified with high accuracy.

### 4.3. Boundary of Method Applicability and Limitation

Previous studies have demonstrated that hyperspectral techniques can achieve the high-accuracy identification of wild and cultivated *Ophiocordyceps sinensis*. Du et al. reported that specimen orientation had no significant influence on hyperspectral identification, with LR achieving accuracies above 98% across different orientations [25]. Similarly, SERS combined with ML achieved an accuracy of 97.99% for wild and cultivated *Ophiocordyceps sinensis* [24]. In comparison, the present study achieved 98.52% accuracy on an independent dataset of 135 real stroma-missing samples using the model trained with intact samples. Although the performance is comparable to previous hyperspectral studies, the main difference is that the present work evaluates model applicability under broken *Ophiocordyceps sinensis* rather than intact sample conditions.

The experimental results indicate that the spectroscopic identification information contribution of the larva part is greater than that of the stroma part. This difference is reflected consistently in both simulated experiments and independent testing. Importantly, the higher accuracy of the intact part over the larva part on real testing suggests that the real damaged samples cannot be fully reproduced by simple segmentation of intact samples. Therefore, the simulation experiments should be regarded as a controlled analysis of part-specific spectroscopic identification information contribution, whereas the independent test on real stroma-missing samples provides the direct evidence for practical generalization.

From a practical perspective, these findings provide guidance for the design of hyperspectral identification instruments for *Ophiocordyceps sinensis*. When the primary objective is to maximize identification accuracy while maintaining applicability to both intact and commonly encountered stroma-missing samples, collecting spectral data from intact samples for model training is preferable. Nevertheless, the current conclusion is limited to samples in which the larva part is substantially preserved. Its applicability to severely fragmented samples, samples with substantial larva loss, or samples measured under substantially different environmental and storage conditions remains to be established.

The wild *Ophiocordyceps sinensis* samples included in this study were exclusively collected from Xizang. Therefore, the wild-sample dataset represents only the Xizang production region, and the applicability of the developed model to wild *Ophiocordyceps sinensis* samples from other major production regions, such as Qinghai, remains to be evaluated. In contrast, the cultivated *Ophiocordyceps sinensis* samples were obtained from multiple commercial brands and production regions, including Qinghai, Zhejiang, and Sichuan, providing relatively broad coverage of commercially available cultivated products. Importantly, Xizang is not a major production region for cultivated *Ophiocordyceps sinensis*. Moreover, the cultivated samples showed a high degree of consistency in the host insect, infecting fungus, and cultivation conditions, thereby limiting variation associated with differences in cultivation background. Taken together, the cultivated dataset provides relatively consistent coverage of the cultivated category, whereas the generalizability of the model to wild *Ophiocordyceps sinensis* samples from other geographic regions remains to be established through further validation using samples from additional major production regions.

In addition, some limitations still exist. First, the anatomical delineation between the larva and stroma parts relied on manual visual segmentation. Future work should achieve automatic extraction. Second, the dataset should encompass samples across broader geographic origins, multiple harvest years, and diverse storage conditions to enhance model generalizability. Third, the current studies have been conducted under laboratory conditions, future research should evaluate and validate in real market environments to ensure model robustness under complex field conditions.

## 5. Conclusions

Based on hyperspectral technology, this study took the identification of wild and cultivated *Ophiocordyceps sinensis* as the research task, evaluated the contribution of the spectroscopic identification information among different parts, and verified the applicability of the trained models in real stroma-missing samples. The main conclusions are as follows:

Both the larva part and the stroma part exhibit strong spectroscopic identification information for identifying wild and cultivated *Ophiocordyceps sinensis*. However, the contribution of the larva part is greater than that of the stroma part. Simultaneously, the intact part can integrate the identification features of both, containing the most abundant identification information. In the simulated datasets, the highest accuracies of the intact part and the larva part reached 100.00%, while the datasets of the stroma part reached 98.91%. Additionally, the mean accuracy of DS_MixILP reached 99.60%, while the mean accuracy of DS_MixILSP decreased to 98.77% when the stroma part was incorporated.

The model using intact *Ophiocordyceps sinensis* identification in previous studies can be directly used to identify broken samples, and there is no need to train the model specifically for stroma-missing samples. Real-scene testing results demonstrate that the model trained based on the intact part dataset still possesses good generalization capability in the real scene. It still maintained an accuracy of 98.52% in the independent test. Meanwhile, the model trained on DS_MixILP maintained an accuracy of 97.04%.

In summary, considering the highest accuracy and the maximum scope of application, the intact part of intact *Ophiocordyceps sinensis* is the optimal detecting part. It can be applied to intact *Ophiocordyceps sinensis* and stroma-missing *Ophiocordyceps sinensis*, and can also achieve high accuracy identification. This study provides a theoretical basis for designing *Ophiocordyceps sinensis* hyperspectral identification instruments for the optimal detection of parts selection.

## Figures and Tables

**Table 1:** The quantity of different types and categories of *Ophiocordyceps sinensis*.

Type	Category	Quantity
Intact *Ophiocordyceps sinensis*	Wild *Ophiocordyceps sinensis*	589
	Cultivated *Ophiocordyceps sinensis*	328
Stroma-missing *Ophiocordyceps sinensis*	Wild *Ophiocordyceps sinensis*	38
	Cultivated *Ophiocordyceps sinensis*	97

**Table 2:** Main parameters of hyperspectral camera.

Parameters	Hyspex VNIR-1024	Hyspex SWIR-384
Spectral range	400–1000 nm	930–2500 nm
Spatial pixel	1024	384
Channel number	108	288
Spectral sampling interval	5.4 nm	5.45 nm

**Table 3:** The quantity of each part in the DS_MixILSP and DS_MixILP during the ten repeated experiments.

Dataset	Part	Experiment Index									
		1	2	3	4	5	6	7	8	9	10
DS_MixILSP	Intact	302	314	298	306	295	301	311	320	333	312
	Larva	295	314	333	296	304	306	302	295	313	308
	Stroma	320	289	286	315	318	310	304	302	271	297
DS_MixILP	Intact	470	486	479	475	486	448	454	447	485	436
	Larva	447	431	438	442	431	469	463	470	432	481

**Table 4:** Hyperparameters for the five ML models.

Model	Hyperparameter	Search Space
LR	penalty	{L1, L2}
	solver	{‘liblinear’, ‘lbfgs’}
	C	{0.001, 0.01, 0.1, 1, 10, 100, 1000, 10,000}
	max_iter	{1000, 10,000}
SVM	kernel	{‘linear’, ‘rbf’}
	C	{0.001, 0.01, 0.1, 1, 10, 100, 1000, 10,000}
	gamma	{0.01, 0.1, 1, ‘scale’}
RF	n_estimators	{100, 200, 300, 500}
	max_depth	{2, 4, 8, 16, None}
	min_samples_split	{2, 4, 8, 16}
	min_samples_leaf	{1, 2, 4}
LightGBM	n_estimators	{100, 300, 500, 700}
	learning_rate	{0.001, 0.01, 0.05, 0.1}
	max_depth	{3, 5, 7, 9}
	num_leaves	{15, 31, 63}
XGBoost	n_estimators	{100, 300, 500, 700}
	learning_rate	{0.001, 0.01, 0.05, 0.1}
	max_depth	{3, 6, 9}
	subsample	{0.7, 0.9, 1}

**Table 5:** Ten-fold cross-validation Acc (%) of different datasets under six models.

Models	DS_IntP	DS_LarP	DS_StrP
FCNN	100.00	99.89	98.15
LR	100.00	100.00	98.91
LightGBM	98.36	97.93	95.53
RF	98.14	97.71	95.09
SVM	100.00	100.00	98.69
XGBoost	98.58	97.60	94.88

**Table 6:** The ten-fold cross-validation Acc (%) of DS_MixILSP in ten experiments on six models.

Experiment Index	Models					
	FCNN	LR	LightGBM	RF	SVM	XGBoost
1	97.93	98.47	94.66	94.44	98.58	94.22
2	97.71	98.91	94.76	94.98	99.35	94.76
3	97.92	98.15	95.31	95.09	98.26	95.53
4	98.80	98.80	95.74	95.42	98.47	94.87
5	97.93	98.69	95.42	93.90	98.47	95.20
6	98.26	99.24	94.33	94.00	98.91	94.55
7	98.25	98.47	95.64	94.76	98.58	95.64
8	97.71	98.80	94.65	93.78	99.02	94.98
9	97.93	99.02	95.09	93.89	99.24	94.66
10	98.03	98.91	95.09	95.31	98.80	95.09
mAcc (%)	98.05 ± 0.32	98.75 ± 0.31	95.07 ± 0.46	94.56 ± 0.63	98.77 ± 0.36	94.95 ± 0.44

**Table 7:** The ten-fold cross-validation Acc (%) of DS_MixILP in ten experiments on six models.

Experiment Index	Models					
	FCNN	LR	LightGBM	RF	SVM	XGBoost
1	99.78	99.78	98.03	97.38	99.78	97.60
2	99.78	99.45	96.95	97.17	99.56	97.28
3	99.78	99.45	97.49	96.84	99.57	96.94
4	100.00	99.67	97.28	96.19	99.35	96.19
5	99.78	99.56	97.60	96.29	99.56	96.29
6	99.67	99.67	97.38	95.86	99.34	96.08
7	99.89	99.78	96.95	96.40	99.67	95.96
8	99.89	99.56	97.16	96.51	99.35	96.95
9	99.67	99.56	96.95	96.73	99.34	96.29
10	99.67	99.56	97.49	96.62	99.67	96.29
mAcc (%)	99.79 ± 0.11	99.60 ± 0.12	97.33 ± 0.35	96.60 ± 0.46	99.52 ± 0.16	96.59 ± 0.56

**Table 8:** The performance indicators of models trained on the single-part datasets when tested on DS_MisStrP.

Trained Models	Acc (%)	Pre (%)	Rec (%)	Spe (%)	F1 (%)
Mod_IntP_FCNN	98.52	95.00	100.00	97.94	97.44
Mod_LarP_FCNN	98.52	95.00	100.00	97.94	97.44
Mod_StrP_FCNN	90.37	100.00	65.79	100.00	79.37
Mod_IntP_LR	98.52	95.00	100.00	97.94	97.44
Mod_LarP_LR	97.04	90.48	100.00	95.88	95.00
Mod_StrP_LR	87.41	100.00	55.26	100.00	71.19
Mod_IntP_SVM	97.78	94.87	97.37	97.94	96.10
Mod_LarP_SVM	97.04	90.48	100.00	95.88	95.00
Mod_StrP_SVM	88.15	100.00	57.89	100.00	73.33

**Table 9:** The Acc (%) of models trained on the mixed-part datasets (DS_MixILSP, DS_MixILP) when tested on DS_MisStrP.

Experiment Index	Mod_MixILSP_FCNN	Mod_MixILP_FCNN	Mod_MixILSP_LR	Mod_MixILP_LR	Mod_MixILSP_SVM	Mod_MixILP_SVM
1	97.04	97.04	95.56	96.30	95.56	96.30
2	95.56	97.78	97.04	97.78	97.78	97.04
3	94.81	97.04	93.33	97.04	96.30	97.04
4	98.52	97.78	92.59	96.30	94.07	96.30
5	95.56	96.30	97.04	97.04	95.56	96.30
6	96.30	96.30	97.78	97.78	97.78	97.78
7	94.81	97.78	95.56	97.78	98.52	97.78
8	96.30	97.04	97.04	96.30	96.30	96.30
9	97.78	97.78	94.81	97.04	97.04	96.30
10	96.30	94.81	95.56	97.04	95.56	97.04
mAcc (%)	96.30 ± 1.21	96.97 ± 0.95	95.63 ± 1.69	97.04 ± 0.60	96.45 ± 1.34	96.81 ± 0.61

## Data Availability

The original contributions presented in this study are included in the article. Further inquiries can be directed to the corresponding authors.

## Appendix A

**Table A1:** Ten-fold cross-validation performance indicators of DS_IntP under six models.

Datasets	Model	Acc (%)	Pre (%)	Rec (%)	Spe (%)	F1 (%)
DS_IntP	FCNN	100.00 ± 0.00	100.00 ± 0.00	100.00 ± 0.00	100.00 ± 0.00	100.00 ± 0.00
	LR	100.00 ± 0.00	100.00 ± 0.00	100.00 ± 0.00	100.00 ± 0.00	100.00 ± 0.00
	LightGBM	98.36 ± 1.18	98.67 ± 1.52	98.81 ± 1.40	97.56 ± 2.81	98.73 ± 0.91
	RF	98.14 ± 1.63	98.33 ± 1.55	98.81 ± 1.80	96.94 ± 2.92	98.56 ± 1.27
	SVM	100.00 ± 0.00	100.00 ± 0.00	100.00 ± 0.00	100.00 ± 0.00	100.00 ± 0.00
	XGBoost	98.58 ± 1.27	98.84 ± 1.56	98.98 ± 1.19	97.85 ± 2.95	98.90 ± 0.97
DS_LarP	FCNN	99.89 ± 0.35	100.00 ± 0.00	99.83 ± 0.54	100.00 ± 0.00	99.91 ± 0.27
	LR	100.00 ± 0.00	100.00 ± 0.00	100.00 ± 0.00	100.00 ± 0.00	100.00 ± 0.00
	LightGBM	97.93 ± 1.81	98.33 ± 1.75	98.47 ± 1.87	96.95 ± 3.21	98.39 ± 1.41
	RF	97.71 ± 1.58	97.82 ± 1.58	98.64 ± 1.34	96.03 ± 2.89	98.23 ± 1.22
	SVM	100.00 ± 0.00	100.00 ± 0.00	100.00 ± 0.00	100.00 ± 0.00	100.00 ± 0.00
	XGBoost	97.60 ± 1.68	98.15 ± 1.66	98.13 ± 1.69	96.64 ± 3.03	98.13 ± 1.31
DS_StrP	FCNN	98.15 ± 1.45	98.67 ± 1.69	98.47 ± 1.87	97.57 ± 3.13	98.56 ± 1.14
	LR	98.91 ± 0.72	99.00 ± 1.16	99.32 ± 0.88	98.17 ± 2.13	99.16 ± 0.56
	LightGBM	95.53 ± 1.19	96.66 ± 2.01	96.43 ± 1.86	93.89 ± 3.84	96.52 ± 0.92
	RF	95.09 ± 2.08	95.64 ± 1.38	96.78 ± 2.32	92.05 ± 2.64	96.20 ± 1.63
	SVM	98.69 ± 0.46	98.99 ± 0.87	98.98 ± 0.88	98.17 ± 1.57	98.98 ± 0.36
	XGBoost	94.88 ± 1.70	96.45 ± 1.78	95.59 ± 2.79	93.60 ± 3.35	95.99 ± 1.36

**Table A2:** Ten-fold cross-validation performance indicators of DS_MixILSP in ten experiments on six models.

Experiment Index	Model	Acc (%)	Pre (%)	Rec (%)	Spe (%)	F1 (%)
1	FCNN	97.93 ± 1.81	98.32 ± 1.58	98.47 ± 1.87	96.96 ± 2.86	98.39 ± 1.42
	LR	98.47 ± 1.47	98.68 ± 1.86	98.98 ± 1.19	97.58 ± 3.44	98.82 ± 1.13
	LightGBM	94.66 ± 1.95	95.32 ± 1.45	96.44 ± 2.32	91.46 ± 2.80	95.86 ± 1.52
	RF	94.44 ± 2.14	94.39 ± 1.56	97.11 ± 2.12	89.63 ± 2.94	95.73 ± 1.66
	SVM	98.58 ± 1.70	98.82 ± 1.39	98.98 ± 1.64	97.87 ± 2.50	98.90 ± 1.34
	XGBoost	94.22 ± 2.05	94.69 ± 1.78	96.43 ± 2.18	90.25 ± 3.43	95.54 ± 1.59
2	FCNN	97.71 ± 1.20	98.19 ± 1.95	98.31 ± 1.38	96.64 ± 3.68	98.23 ± 0.92
	LR	98.91 ± 1.36	99.01 ± 1.76	99.32 ± 0.88	98.18 ± 3.26	99.16 ± 1.04
	LightGBM	94.76 ± 2.91	96.00 ± 2.96	95.93 ± 2.90	92.66 ± 5.69	95.93 ± 2.23
	RF	94.98 ± 2.43	95.23 ± 2.57	97.12 ± 2.27	91.14 ± 4.95	96.14 ± 1.84
	SVM	99.35 ± 1.38	99.34 ± 1.59	99.66 ± 0.71	98.79 ± 2.93	99.50 ± 1.06
	XGBoost	94.76 ± 3.30	95.64 ± 2.60	96.27 ± 3.37	92.05 ± 4.91	95.93 ± 2.56
3	FCNN	97.92 ± 2.03	98.35 ± 2.02	98.46 ± 2.48	96.92 ± 3.88	98.38 ± 1.58
	LR	98.15 ± 1.27	98.19 ± 1.80	98.98 ± 1.20	96.64 ± 3.39	98.57 ± 0.97
	LightGBM	95.31 ± 2.66	96.04 ± 2.97	96.77 ± 2.18	92.67 ± 5.78	96.38 ± 2.01
	RF	95.09 ± 2.00	95.74 ± 2.99	96.77 ± 1.50	92.06 ± 5.98	96.22 ± 1.46
	SVM	98.26 ± 1.05	98.34 ± 1.35	98.98 ± 1.20	96.95 ± 2.47	98.65 ± 0.81
	XGBoost	95.53 ± 1.95	96.19 ± 2.47	96.94 ± 1.56	92.97 ± 4.83	96.55 ± 1.47
4	FCNN	98.80 ± 1.31	99.16 ± 1.19	98.98 ± 1.43	98.46 ± 2.20	99.07 ± 1.02
	LR	98.80 ± 0.96	99.16 ± 0.89	98.98 ± 1.19	98.47 ± 1.62	99.06 ± 0.75
	LightGBM	95.74 ± 1.51	96.83 ± 2.42	96.60 ± 0.80	94.18 ± 4.73	96.70 ± 1.11
	RF	95.42 ± 1.91	95.40 ± 2.25	97.62 ± 1.64	91.44 ± 4.33	96.48 ± 1.46
	SVM	98.47 ± 0.76	98.82 ± 1.12	98.81 ± 0.82	97.86 ± 2.06	98.81 ± 0.59
	XGBoost	94.87 ± 1.99	95.68 ± 2.52	96.44 ± 1.68	92.05 ± 4.85	96.04 ± 1.51
5	FCNN	97.93 ± 1.20	98.31 ± 1.11	98.47 ± 1.25	96.94 ± 2.05	98.39 ± 0.94
	LR	98.69 ± 1.00	99.16 ± 0.89	98.81 ± 1.14	98.47 ± 1.62	98.98 ± 0.78
	LightGBM	95.42 ± 2.10	95.83 ± 1.58	97.12 ± 2.40	92.37 ± 2.98	96.46 ± 1.66
	RF	93.90 ± 2.13	94.40 ± 2.45	96.27 ± 1.75	89.63 ± 4.87	95.30 ± 1.62
	SVM	98.47 ± 1.05	99.00 ± 1.57	98.64 ± 1.07	98.17 ± 2.93	98.81 ± 0.81
	XGBoost	95.20 ± 2.06	95.67 ± 1.86	96.95 ± 2.37	92.06 ± 3.60	96.29 ± 1.61
6	FCNN	98.26 ± 1.55	98.65 ± 1.31	98.64 ± 1.75	97.57 ± 2.39	98.64 ± 1.22
	LR	99.24 ± 0.90	99.49 ± 0.81	99.32 ± 0.88	99.08 ± 1.48	99.41 ± 0.70
	LightGBM	94.33 ± 1.24	95.30 ± 1.47	95.92 ± 1.44	91.46 ± 2.80	95.60 ± 0.97
	RF	94.00 ± 2.13	93.80 ± 2.06	97.11 ± 2.12	88.41 ± 4.02	95.41 ± 1.64
	SVM	98.91 ± 1.03	99.00 ± 1.16	99.32 ± 1.19	98.16 ± 2.16	99.15 ± 0.80
	XGBoost	94.55 ± 1.98	95.46 ± 1.58	96.10 ± 2.53	91.77 ± 2.87	95.76 ± 1.57
7	FCNN	98.25 ± 1.48	98.82 ± 1.13	98.47 ± 2.03	97.87 ± 2.05	98.63 ± 1.16
	LR	98.47 ± 1.28	98.68 ± 1.70	98.98 ± 1.19	97.57 ± 3.13	98.82 ± 0.98
	LightGBM	95.64 ± 1.36	96.20 ± 2.21	97.11 ± 1.97	92.98 ± 4.40	96.62 ± 1.05
	RF	94.76 ± 2.56	94.83 ± 2.94	97.28 ± 3.68	90.23 ± 6.11	95.97 ± 2.01
	SVM	98.58 ± 1.36	98.84 ± 1.74	98.98 ± 1.19	97.86 ± 3.24	98.90 ± 1.05
	XGBoost	95.64 ± 1.53	96.49 ± 1.76	96.77 ± 2.46	93.60 ± 3.41	96.60 ± 1.23
8	FCNN	97.71 ± 1.41	98.49 ± 1.22	97.97 ± 2.08	97.26 ± 2.24	98.21 ± 1.11
	LR	98.80 ± 1.08	98.67 ± 1.30	99.49 ± 0.82	97.57 ± 2.39	99.07 ± 0.84
	LightGBM	94.65 ± 2.03	95.58 ± 2.93	96.26 ± 3.09	91.78 ± 5.88	95.86 ± 1.58
	RF	93.78 ± 3.28	93.63 ± 4.90	97.28 ± 2.15	87.52 ± 10.81	95.32 ± 2.27
	SVM	99.02 ± 0.81	98.99 ± 0.87	99.49 ± 0.82	98.17 ± 1.57	99.24 ± 0.63
	XGBoost	94.98 ± 1.93	95.20 ± 3.52	97.28 ± 2.17	90.84 ± 7.16	96.16 ± 1.41
9	FCNN	97.93 ± 1.58	98.49 ± 1.46	98.30 ± 1.79	97.26 ± 2.65	98.38 ± 1.23
	LR	99.02 ± 0.95	99.00 ± 1.16	99.49 ± 0.82	98.18 ± 2.12	99.24 ± 0.74
	LightGBM	95.09 ± 2.69	96.14 ± 2.15	96.27 ± 3.55	93.00 ± 4.04	96.16 ± 2.15
	RF	93.89 ± 4.02	94.28 ± 3.71	96.43 ± 3.70	89.37 ± 7.15	95.30 ± 3.06
	SVM	99.24 ± 0.90	99.17 ± 1.17	99.66 ± 0.71	98.48 ± 2.14	99.41 ± 0.69
	XGBoost	94.66 ± 2.75	95.67 ± 2.55	96.10 ± 3.58	92.08 ± 4.77	95.84 ± 2.18
10	FCNN	98.03 ± 1.71	98.49 ± 1.48	98.47 ± 1.69	97.23 ± 2.71	98.47 ± 1.32
	LR	98.91 ± 1.15	98.84 ± 1.36	99.49 ± 0.82	97.86 ± 2.53	99.16 ± 0.89
	LightGBM	95.09 ± 2.87	96.13 ± 2.05	96.27 ± 3.81	92.98 ± 3.81	96.16 ± 2.32
	RF	95.31 ± 2.67	95.21 ± 1.98	97.62 ± 2.91	91.15 ± 3.67	96.38 ± 2.09
	SVM	98.80 ± 0.96	98.99 ± 0.87	99.15 ± 1.20	98.17 ± 1.57	99.07 ± 0.75
	XGBoost	95.09 ± 2.91	95.97 ± 2.10	96.44 ± 3.95	92.68 ± 3.84	96.16 ± 2.35

**Table A3:** Ten-fold cross-validation performance indicators of DS_MixILP in ten experiments on six models.

Experiment Index	Model	Acc (%)	Pre (%)	Rec (%)	Spe (%)	F1 (%)
1	FCNN	99.78 ± 0.46	99.83 ± 0.53	99.83 ± 0.54	99.70 ± 0.96	99.83 ± 0.36
	LR	99.78 ± 0.46	100.00 ± 0.00	99.66 ± 0.71	100.00 ± 0.00	99.83 ± 0.36
	LightGBM	98.03 ± 0.87	99.01 ± 1.40	97.96 ± 1.76	98.16 ± 2.59	98.46 ± 0.68
	RF	97.38 ± 1.17	97.85 ± 1.89	98.13 ± 1.48	96.03 ± 3.52	97.97 ± 0.90
	SVM	99.78 ± 0.46	100.00 ± 0.00	99.66 ± 0.71	100.00 ± 0.00	99.83 ± 0.36
	XGBoost	97.60 ± 1.23	98.35 ± 1.90	97.96 ± 1.76	96.95 ± 3.52	98.13 ± 0.95
2	FCNN	99.78 ± 0.46	99.83 ± 0.53	99.83 ± 0.54	99.70 ± 0.96	99.83 ± 0.36
	LR	99.45 ± 0.77	99.66 ± 0.71	99.49 ± 0.82	99.39 ± 1.28	99.57 ± 0.61
	LightGBM	96.95 ± 1.68	98.64 ± 1.32	96.60 ± 2.88	97.56 ± 2.40	97.58 ± 1.38
	RF	97.17 ± 1.05	97.84 ± 1.70	97.79 ± 1.97	96.04 ± 3.20	97.79 ± 0.83
	SVM	99.56 ± 0.56	99.50 ± 0.81	99.83 ± 0.55	99.08 ± 1.48	99.66 ± 0.44
	XGBoost	97.28 ± 1.64	98.31 ± 1.09	97.45 ± 3.02	96.95 ± 2.02	97.85 ± 1.34
3	FCNN	99.78 ± 0.46	99.83 ± 0.53	99.83 ± 0.54	99.70 ± 0.96	99.83 ± 0.36
	LR	99.45 ± 0.77	99.66 ± 0.71	99.49 ± 0.82	99.38 ± 1.30	99.58 ± 0.60
	LightGBM	97.49 ± 1.46	98.18 ± 1.80	97.97 ± 2.37	96.63 ± 3.37	98.04 ± 1.15
	RF	96.84 ± 0.96	97.20 ± 2.03	97.96 ± 1.75	94.81 ± 3.82	97.55 ± 0.73
	SVM	99.57 ± 0.76	99.83 ± 0.53	99.49 ± 1.14	99.70 ± 0.96	99.66 ± 0.60
	XGBoost	96.94 ± 1.24	97.65 ± 1.40	97.62 ± 2.29	95.72 ± 2.59	97.61 ± 0.99
4	FCNN	100.00 ± 0.00	100.00 ± 0.00	100.00 ± 0.00	100.00 ± 0.00	100.00 ± 0.00
	LR	99.67 ± 0.53	99.83 ± 0.53	99.66 ± 0.71	99.70 ± 0.96	99.75 ± 0.41
	LightGBM	97.28 ± 1.64	98.47 ± 1.24	97.29 ± 1.99	97.27 ± 2.24	97.86 ± 1.30
	RF	96.19 ± 2.06	96.39 ± 2.34	97.79 ± 2.66	93.30 ± 4.46	97.05 ± 1.60
	SVM	99.35 ± 0.56	99.67 ± 0.70	99.32 ± 0.88	99.39 ± 1.28	99.49 ± 0.44
	XGBoost	96.19 ± 2.24	97.47 ± 1.91	96.61 ± 2.88	95.44 ± 3.56	97.01 ± 1.79
5	FCNN	99.78 ± 0.46	99.83 ± 0.53	99.83 ± 0.54	99.70 ± 0.96	99.83 ± 0.36
	LR	99.56 ± 0.56	99.67 ± 0.70	99.66 ± 0.72	99.39 ± 1.28	99.66 ± 0.44
	LightGBM	97.60 ± 1.44	98.16 ± 1.63	98.13 ± 1.49	96.64 ± 3.03	98.13 ± 1.12
	RF	96.29 ± 2.00	96.82 ± 1.82	97.45 ± 2.44	94.20 ± 3.36	97.11 ± 1.57
	SVM	99.56 ± 0.77	99.83 ± 0.53	99.49 ± 1.16	99.70 ± 0.96	99.66 ± 0.61
	XGBoost	96.29 ± 1.06	97.32 ± 1.55	96.94 ± 2.38	95.11 ± 2.97	97.10 ± 0.85
6	FCNN	99.67 ± 0.53	99.83 ± 0.53	99.66 ± 0.71	99.70 ± 0.96	99.75 ± 0.41
	LR	99.67 ± 0.53	99.83 ± 0.53	99.66 ± 0.71	99.70 ± 0.96	99.75 ± 0.41
	LightGBM	97.38 ± 1.28	98.32 ± 1.57	97.62 ± 1.43	96.93 ± 2.90	97.96 ± 1.00
	RF	95.86 ± 2.10	96.53 ± 2.49	97.11 ± 2.27	93.60 ± 4.63	96.79 ± 1.62
	SVM	99.34 ± 0.92	99.66 ± 0.71	99.32 ± 1.19	99.38 ± 1.30	99.49 ± 0.72
	XGBoost	96.08 ± 1.79	97.48 ± 1.78	96.43 ± 2.82	95.42 ± 3.29	96.92 ± 1.45
7	FCNN	99.89 ± 0.34	99.83 ± 0.53	100.00 ± 0.00	99.70 ± 0.96	99.92 ± 0.27
	LR	99.78 ± 0.46	100.00 ± 0.00	99.66 ± 0.71	100.00 ± 0.00	99.83 ± 0.36
	LightGBM	96.95 ± 1.34	97.82 ± 1.73	97.45 ± 1.20	96.04 ± 3.20	97.62 ± 1.04
	RF	96.40 ± 1.92	96.41 ± 2.74	98.13 ± 0.97	93.30 ± 5.30	97.24 ± 1.43
	SVM	99.67 ± 0.53	99.83 ± 0.53	99.66 ± 0.71	99.70 ± 0.96	99.75 ± 0.41
	XGBoost	95.96 ± 2.31	96.99 ± 2.40	96.77 ± 2.62	94.53 ± 4.48	96.85 ± 1.82
8	FCNN	99.89 ± 0.34	99.83 ± 0.53	100.00 ± 0.00	99.70 ± 0.96	99.92 ± 0.27
	LR	99.56 ± 0.56	99.83 ± 0.53	99.49 ± 0.82	99.70 ± 0.96	99.66 ± 0.44
	LightGBM	97.16 ± 1.65	98.16 ± 1.96	97.45 ± 1.65	96.63 ± 3.73	97.79 ± 1.26
	RF	96.51 ± 1.43	96.70 ± 1.86	97.96 ± 2.08	93.91 ± 3.49	97.30 ± 1.11
	SVM	99.35 ± 0.56	99.33 ± 0.86	99.66 ± 0.71	98.78 ± 1.58	99.49 ± 0.44
	XGBoost	96.95 ± 1.61	97.97 ± 1.53	97.28 ± 2.15	96.34 ± 2.80	97.61 ± 1.27
9	FCNN	99.67 ± 0.53	99.67 ± 0.70	99.83 ± 0.55	99.39 ± 1.28	99.74 ± 0.41
	LR	99.56 ± 0.77	99.66 ± 0.71	99.66 ± 0.72	99.38 ± 1.30	99.66 ± 0.59
	LightGBM	96.95 ± 1.52	97.68 ± 2.02	97.62 ± 2.14	95.72 ± 3.87	97.62 ± 1.19
	RF	96.73 ± 1.15	97.21 ± 2.28	97.79 ± 1.96	94.81 ± 4.32	97.47 ± 0.88
	SVM	99.34 ± 0.92	99.83 ± 0.54	99.15 ± 1.20	99.69 ± 0.99	99.49 ± 0.72
	XGBoost	96.29 ± 1.28	97.18 ± 1.99	97.11 ± 2.12	94.80 ± 3.83	97.11 ± 1.00
10	FCNN	99.67 ± 0.73	99.83 ± 0.53	99.66 ± 1.07	99.70 ± 0.96	99.74 ± 0.58
	LR	99.56 ± 0.56	99.67 ± 0.70	99.66 ± 0.72	99.38 ± 1.30	99.66 ± 0.44
	LightGBM	97.49 ± 1.70	98.48 ± 1.46	97.62 ± 2.15	97.24 ± 2.68	98.03 ± 1.35
	RF	96.62 ± 1.58	97.39 ± 2.70	97.45 ± 1.84	95.10 ± 5.25	97.38 ± 1.19
	SVM	99.67 ± 0.53	99.67 ± 0.70	99.83 ± 0.54	99.38 ± 1.30	99.75 ± 0.41
	XGBoost	96.29 ± 2.42	97.33 ± 2.48	96.94 ± 2.75	95.10 ± 4.63	97.11 ± 1.90

**Table A4:** The performance indicators of models trained on the mixed-part datasets (DS_MixILSP) when tested on DS_MisStrP.

Experiment Index	Model	Acc (%)	Pre (%)	Rec (%)	Spe (%)	F1 (%)
1	FCNN	97.04	97.22	92.11	98.97	94.59
	LR	95.56	92.11	92.11	96.91	92.11
	SVM	95.56	88.10	97.37	94.85	92.50
2	FCNN	95.56	97.06	86.84	98.97	91.67
	LR	97.04	94.74	94.74	97.94	94.74
	SVM	96.30	90.24	97.37	95.88	93.67
3	FCNN	94.81	89.74	92.11	95.88	90.91
	LR	93.33	87.18	89.47	94.85	88.31
	SVM	94.07	84.09	97.37	92.78	90.24
4	FCNN	98.52	95.00	100.00	97.94	97.44
	LR	92.59	85.00	89.47	93.81	87.18
	SVM	95.56	88.10	97.37	94.85	92.50
5	FCNN	95.56	97.06	86.84	98.97	91.67
	LR	97.04	97.22	92.11	98.97	94.59
	SVM	97.78	94.87	97.37	97.94	96.10
6	FCNN	96.30	97.14	89.47	98.97	93.15
	LR	97.78	94.87	97.37	97.94	96.10
	SVM	98.52	95.00	100.00	97.94	97.44
7	FCNN	94.81	96.97	84.21	98.97	90.14
	LR	95.56	90.00	94.74	95.88	92.31
	SVM	96.30	90.24	97.37	95.88	93.67
8	FCNN	96.30	97.14	89.47	98.97	93.15
	LR	97.04	92.50	97.37	96.91	94.87
	SVM	97.04	92.50	97.37	96.91	94.87
9	FCNN	97.78	97.30	94.74	98.97	96.00
	LR	94.81	87.80	94.74	94.85	91.14
	SVM	95.56	88.10	97.37	94.85	92.50
10	FCNN	96.30	97.14	89.47	98.97	93.15
	LR	95.56	94.44	89.47	97.94	91.89
	SVM	97.78	94.87	97.37	97.94	96.10

**Table A5:** The performance indicators of models trained on the mixed-part datasets (DS_MixILP) when tested on DS_MisStrP.

Experiment Index	Model	Acc (%)	Pre (%)	Rec (%)	Spe (%)	F1 (%)
1	FCNN	97.04	92.50	97.37	96.91	94.87
	LR	96.30	88.37	100.00	94.85	93.83
	SVM	96.30	88.37	100.00	94.85	93.83
2	FCNN	97.78	94.87	97.37	97.94	96.10
	LR	97.78	92.68	100.00	96.91	96.20
	SVM	97.04	90.48	100.00	95.88	95.00
3	FCNN	97.04	92.50	97.37	96.91	94.87
	LR	97.04	90.48	100.00	95.88	95.00
	SVM	97.04	90.48	100.00	95.88	95.00
4	FCNN	97.78	92.68	100.00	96.91	96.20
	LR	96.30	88.37	100.00	94.85	93.83
	SVM	96.30	88.37	100.00	94.85	93.83
5	FCNN	96.30	90.24	97.37	95.88	93.67
	LR	97.04	90.48	100.00	95.88	95.00
	SVM	96.30	88.37	100.00	94.85	93.83
6	FCNN	96.30	90.24	97.37	95.88	93.67
	LR	97.78	92.68	100.00	96.91	96.20
	SVM	97.78	92.68	100.00	96.91	96.20
7	FCNN	97.78	92.68	100.00	96.91	96.20
	LR	97.78	92.68	100.00	96.91	96.20
	SVM	97.78	92.68	100.00	96.91	96.20
8	FCNN	97.04	92.50	97.37	96.91	94.87
	LR	96.30	88.37	100.00	94.85	93.83
	SVM	96.30	88.37	100.00	94.85	93.83
9	FCNN	97.78	94.87	97.37	97.94	96.10
	LR	97.04	90.48	100.00	95.88	95.00
	SVM	96.30	88.37	100.00	94.85	93.83
10	FCNN	94.81	86.05	97.37	93.81	91.36
	LR	97.04	90.48	100.00	95.88	95.00
	SVM	97.04	90.48	100.00	95.88	95.00
